# Computational redesign of Beta-27 Fab with substantially better predicted binding affinity to the SARS-CoV-2 Omicron variant than human ACE2 receptor

**DOI:** 10.1038/s41598-023-42442-1

**Published:** 2023-09-19

**Authors:** Wantanee Treewattanawong, Thassanai Sitthiyotha, Surasak Chunsrivirot

**Affiliations:** Structural and Computational Biology Research Unit, Department of Biochemistry, Faculty of Science, Chulalongkorn University, Pathumwan, Bangkok, 10330 Thailand

**Keywords:** Protein design, Computational chemistry

## Abstract

During the COVID-19 pandemic, SARS-CoV-2 has caused large numbers of morbidity and mortality, and the Omicron variant (B.1.1.529) was an important variant of concern. To enter human cells, the receptor-binding domain (RBD) of the S1 subunit of SARS-CoV-2 (SARS-CoV-2-RBD) binds to the peptidase domain (PD) of Angiotensin-converting enzyme 2 (ACE2) receptor. Disrupting the binding interactions between SARS-CoV-2-RBD and ACE2-PD using neutralizing antibodies is an effective COVID-19 therapeutic solution. Previous study found that Beta-27 Fab, which was obtained by digesting the full IgG antibodies that were isolated from a patient infected with SARS-CoV-2 Beta variant, can neutralize Victoria, Alpha (B.1.1.7), Beta (B.1.351), Gamma (P.1), and Delta (B.1.617.2) variants. This study employed computational protein design and molecular dynamics (MD) to investigate and enhance the binding affinity of Beta-27 Fab to SARS-CoV-2-RBD Omicron variant. MD results show that five best designed Beta-27 Fabs (Beta-27-D01 Fab, Beta-27-D03 Fab, Beta-27-D06 Fab, Beta-27-D09 Fab and Beta-27-D10 Fab) were predicted to bind to Omicron RBD in the area, where ACE2 binds, with significantly better binding affinities than Beta-27 Fab and ACE2. Their enhanced binding affinities are mostly caused by increased binding interactions of CDR L2 and L3. They are promising candidates that could potentially be employed to disrupt the binding between ACE2 and Omicron RBD.

## Introduction

The global pandemic coronavirus disease 2019 (COVID-19) caused by severe acute respiratory syndrome coronavirus 2 (SARS-CoV-2) has caused large numbers of global cases and deaths^1–7^. Since its first discovery in Wuhan city of China^7–9^, multiple variants of concern (VOCs), including Alpha (B.1.1.7), Beta (B.1.351), Gamma (P.1), Delta (B.1.617.2), and Omicron (B.1.1.529)^10^ have emerged. SARS-CoV-2 and VOCs contain four main structural proteins consisting of nucleocapsid (N) protein, membrane (M) protein, envelope (E) protein, and spike (S) protein^3,11–14^. The Spike (S) protein consists of two functional subunits including receptor binding S1 and membrane fusion S2 subunits^15–18^. SARS-CoV-2 enters human cells by two key processes: 1) the binding between the receptor-binding domain (RBD) of the S1 subunit and the peptidase domain (PD) of the Angiotensin-converting enzyme 2 (ACE2) that is the receptor for SARS-CoV-2 of human cells, and 2) the fusion between a viral membrane and the host-cellular membranes through the S2 subunit^3,5,12,19,20^. Previous study found that monomeric human ACE2 bound to the Omicron and wild-type RBD with the dissociation constant (*K*_*D*_) of 38.9 ± 10.5 nM and 75.5 ± 2.1 nM, respectively^21^.

To prevent the entry of SARS-CoV-2 into human cells, blocking the binding between SARS-CoV-2-RBD and ACE2-PD is a promising therapeutic strategy. Various potential therapeutic solutions such as peptide inhibitors, small-molecule drugs, and neutralizing antibodies have been widely investigated**,** and they can be used to disrupt the binding between SARS-CoV-2-RBD and ACE2^4,22–29^. One of the effective therapeutic solutions to control COVID-19 epidemic is neutralizing antibodies because they can effectively inhibit COVID-19 infection of human cells by blocking the binding between ACE2 and SARS-CoV-2-RBD. The U.S. Food and Drug Administration (FDA) has approved some neutralizing antibodies, such as sotrovimab^30,31^, REGEN-COV (casirivimab and imdevimab)^32–35^, the combination of bamlanivimab and etesevimab^33,35^, EVUSHELD (the co-packaging of tixagevimab and cilgavimab)^36,37^, and bebtelovimab^38^ for emergency use authorization (EUA) to treat mild-to-moderate COVID-19 in adults and pediatric patients. However, they are not effective or no longer authorized for the treatment of COVID-19 due to the Omicron variant^37,39–42^. Recently, neutralizing antibody named ACTEMRA (tocilizumab)^43^ has already been given EUA for current emergency use by FDA.

The previous experimental study found that the Fab fragment of Beta-27 (Beta-27 Fab), which was obtained by digesting the full IgG antibodies that were isolated from a patient infected with SARS-CoV-2 Beta variant, can neutralize Victoria, Alpha, Beta, Gamma, and Delta variants (IC_50_ of ~ 0.018 ± 0.002, 0.018 ± 0.000, 0.009 ± 0.000, 0.006 ± 0.002 and 0.021 ± 0.004 μg/ml, respectively)^44^. Since Beta-27 Fab can neutralize various variants, we hypothesized that it may be able to neutralize the Omicron variant as well. However, the knowledge on the binding between Beta-27 Fab and SARS-CoV-2-RBD Omicron variant is limited.

Computational techniques have been used to develop COVID-19 potential protein therapeutic solutions including peptide inhibitors and antibodies. In terms of peptide inhibitors, we employed computational protein design (Rosetta) and MD (AMBER) to design 25 mer-peptide binders (SPB25) of SARS-CoV-2-RBD with better predicted binding affinity than 23-mer peptide binder (SBP1)^45^, the experimentally proven inhibitor of SARS-CoV-2-RBD, and ACE2^46^. In terms of antibodies, we employed computational protein design (RosettaAntibodyDesign; RAbD) and MD to redesign the Fab fragment of CC12.3 (Fab CC12.3), and our three best designed Fabs CC12.3 (CC12.3-D02, CC12.3-D05, and CC12.3-D08) have better predicted binding affinity to SARS-CoV-2-RBD than that of Fab CC12.3 and ACE2^47^. Furthermore, Rangel et al. employed a fragment-based computational design approach to design antibodies targeting SARS-CoV-2-RBD. They found that all designed antibodies are highly stable and bound to their targets with nanomolar affinities^48^. Moreover, Chen et al. used virtual scanning mutagenesis and MD to improve the binding affinity to SARS-CoV-2-RBD of P2B-2F6, which was isolated from single B cells of SARS-CoV-2 infected patients. Their experimental results show that two P2B-2F6 mutants (H:V106R and H:V106R/H:P107Y) have binding affinities to SARS-CoV-2-RBD better than P2B-2F6 and other mutants^13^. Additionally, Shariatifar et al. employed MD to design two antibodies (V1 and V2) based on CR3022 that were predicted to bind to SARS-CoV2-RBD better than CR3022^49^. Additionally, using computational antibody design and experimental affinity enhancement, Jeong et al. designed D27LEY that bound to RBDs of various SARS-CoV2 variants with picomolar binding affinities^50^.

To investigate and increase the binding affinity of Beta-27 Fab to Omicron RBD so that its binding affinity is substantially better than human ACE2 and Beta-27 Fab, we employed computational protein design (RAbD) and MD (AMBER) in this study. Using the complex structure of Beta-27 Fab and Omicron RBD, which was constructed by modifying the crystal structure of Beta-27 Fab/Beta RBD complex (PDB code: 7PS1^44^), as a design template, we redesigned all complementarity-determining regions (CDRs) H1, H2, H3, L1, L2 and L3 of Beta-27 Fab. The designed Beta-27 Fabs with enhanced predicted binding affinities to Omicron RBD are promising candidates that could potentially be used to disrupt the binding between ACE2 and Omicron RBD.

## Results

### Computational design of Beta-27 Fab

The structure of Beta-27 Fab/Omicron RBD complex, which was constructed by modifying the crystal structure of Beta-27 Fab/Beta RBD complex (PDB code: 7PS1^44^), was used as a designed template. We employed RAbD^51^ to redesign CDRs H1, H2, H3, L1, L2 and L3 of Beta-27 Fab to enhance the binding affinity of Beta-27 Fab to Omicron RBD so that its binding affinity is better than Beta-27 Fab and ACE2. Each residue of all CDRs was allowed to be any of standard amino acids. As shown in Table 1, the top ten best ΔG_bind (Rosetta)_ values of the designed Beta-27 Fabs (Beta-27-D01 to Beta-27-D10 Fabs) were selected for MD to validate whether their predicted binding affinities by the more accurate molecular mechanics–generalized born surface area (MM-GBSA) method^52–54^ (ΔG_bind (MM-GBSA)_) were better than that of Beta-27 Fab (ΔΔG_bind (MM-GBSA)_ < 0 kcal/mol).

**Table 1:** Predicted binding free energies (ΔG_bind (Rosetta)_) of designed Beta-27 Fabs to SARS-CoV-2-RBD Omicron variant and their CDR sequences. The mutated residues are underlined.

System	ΔG_bind (Rosetta)_ (REU)	Heavy chain			Light chain		
		CDR H1 (23–35)	CDR H2 (50–58)	CDR H3 (96–106)	CDR L1 (24–35)	CDR L2 (50–57)	CDR L3 (90–97)
Beta-27	–	AASGLTVRSNYMN	LIYSGGSTF	ARDLVVYGMDV	RASQSVSSSSLA	YGTSSRAT	QQYGSSPL
Beta-27-D01	− 51.26	TASGFILSRTWLT	LISSSGTTF	ARLLGYLGMDV	KFSEAVIYLFFC	YSTSYLYP	MYYTQVPY
Beta-27-D02	− 49.80	VVLGLNISYNWMS	IIWSGGTTY	ARLLNYLGMDV	KFSEAVLFLLVC	YETYKLQS	VFWTLVPY
Beta-27-D03	− 49.01	VASGLNLSANWWT	LISSSGTTF	ARLLGVLGMDV	QFSEAVIFLYVA	YETSKLYP	VFYTQVPY
Beta-27-D04	− 47.66	TASGLNISYNWMT	LIYSSGTTY	ARLLGYLGMDV	KFSEAVLYLQFC	YETSKLYP	CFFGEVPV
Beta-27-D05	− 46.89	KASGFTVSSTYMN	LISSGGTTF	ARLLGMDGMDV	KFSEAVSQLYVC	YQTSKLHP	MFYTQVPI
Beta-27-D06	− 46.81	IVSGLDISYTVMT	VIFSSGTTY	ARLLGYLGMDV	KSSEAVMQIYVA	YATTYLAP	CLYGEVPY
Beta-27-D07	− 46.54	TVSGFNISYTWMT	LIYSSGTTY	ARLLGMDGMDV	QFSEAVVFLYVC	YQTYILHP	MFMTEVPI
Beta-27-D08	− 46.45	VASGLNVSKNWLS	LIYSSGTTY	ARLLNYLGMDV	KSSEAVLYLLFL	YATSILAP	CFIGEVPV
Beta-27-D09	− 46.43	TASGLNISYNWMN	LIYSSGTTY	ARLLNVYGMDV	KSSEAIVFIYWC	YDTSLLHP	LMIGEVPQ
Beta-27-D10	− 46.26	TASGLVVSSNWLS	LIYSSGTTF	ARLLGYLGMDV	KFSEAILYLIVC	YETSKLHE	VMFTEVPY

### Validation by MD

**Figure 1:**
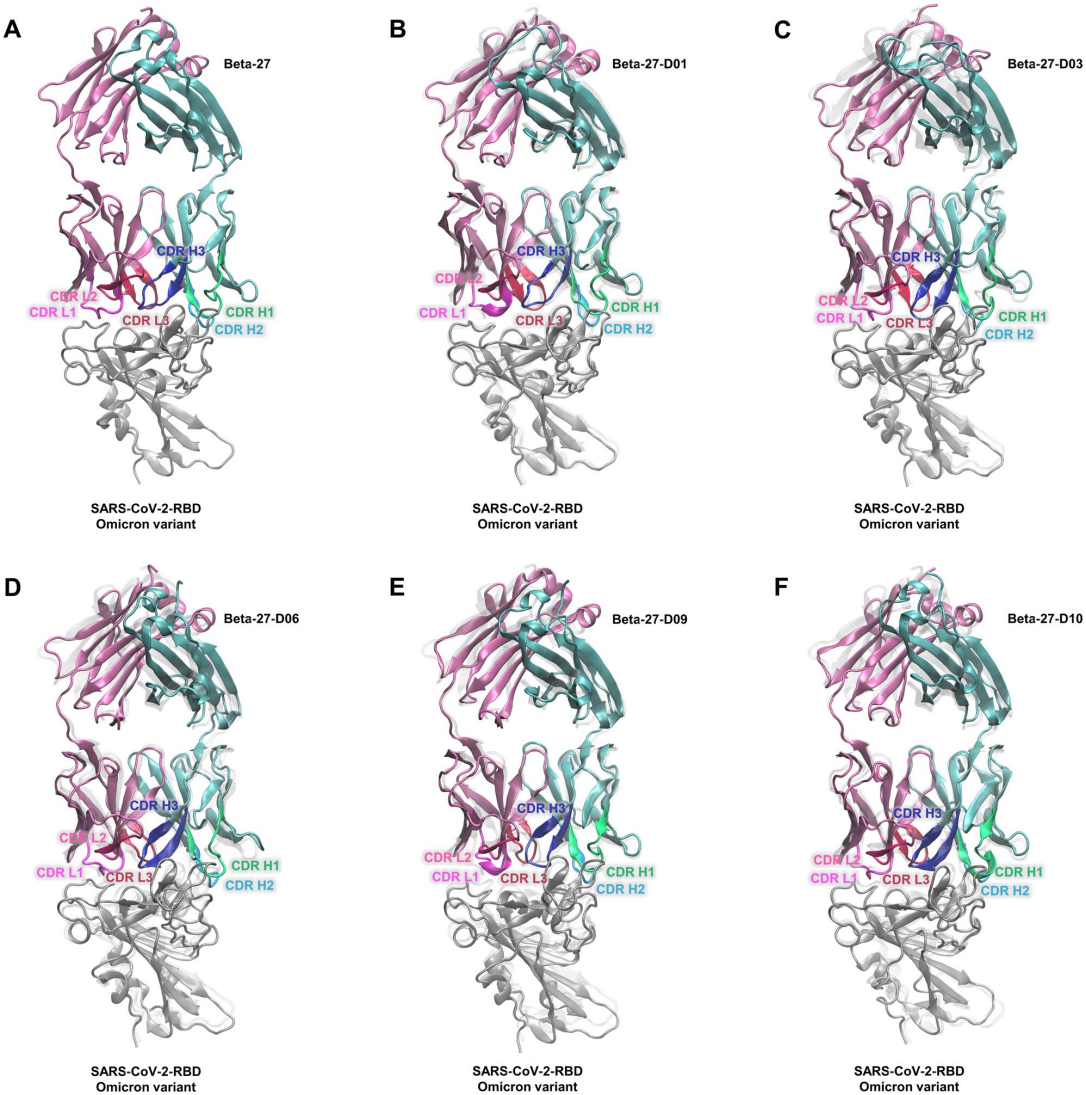
Overall structures of the heavy chain (light blue) and the light chain (light pink) of (**A**) Beta-27, (**B**) Beta-27-D01, (**C**) Beta-27-D03, (**D**) Beta-27-D06, (**E**) Beta-27-D09, and (**F**) Beta-27-D10 Fabs binding to SARS-CoV-2-RBD Omicron variant (gray). CDRs H1, H2, H3, L1, L2, and L3 are colored in green, blue, dark blue, magenta, hot pink, and red, respectively. The designed Beta-27 Fabs/SARS-CoV-2-RBD Omicron variant complexes were superimposed with Beta-27 Fab/SARS-CoV-2-RBD Omicron variant complex (light gray).

MD was performed on structures of Beta-27 and designed Beta-27 Fabs with the top ten best ΔG_bind (Rosetta)_ in complex with Omicron RBD. To analyze structural stabilities, root mean square deviation (RMSD) values of all atoms and backbone atoms were calculated, using residue 1 to 115 of the heavy chain, residue 1 to 106 of the light chain of Beta-27 and designed Beta-27 Fabs (these domains are involved in binding to Omicron RBD), and all residues of Omicron RBD. As show in RMSD plots in Figure S1, all systems are likely to be stable in the range of 80–100 ns; thus, 80–100 ns trajectories were chosen for further analyses. To predict the binding affinities, ΔG_bind (MM-GBSA)_ of all systems during the 80–100 ns trajectories were computed by the MM-GBSA method. Table 2 shows that ΔG_bind (MM-GBSA)_ of the Beta-27 Fab/Omicron RBD complex is − 100.8 ± 0.3 kcal/mol. Five of ten designed Beta-27 Fabs including Beta-27-D01, Beta-27-D03, Beta-27-D06, Beta-27-D09, and Beta-27-D10 Fabs have better ΔG_bind (MM-GBSA)_ than Beta-27 Fab with ΔΔG_bind (MM-GBSA)_ of − 10.4 ± 0.5, − 14.3 ± 0.5, − 8.5 ± 0.5, − 13.1 ± 0.6, and − 19.9 ± 0.8 kcal/mol, respectively. Their predicted binding affinities are also better than that of ACE2. As shown in Fig. 1, overall binding poses of Beta-27-D01, Beta-27-D03, Beta-27-D06, Beta-27-D09, and Beta-27-D10 Fabs have the binding positions and orientations relatively similar to Beta-27 Fab.

**Table 2:** The binding free energies of ACE2, Beta-27 Fab and designed Beta-27 Fabs to SARS-CoV-2-RBD Omicron variant, as calculated by Rosetta and MM-GBSA method.

System	ΔG_bind (Rosetta)_ (REU)	ΔG_bind (MM-GBSA)_ (kcal/mol)	ΔΔG_bind (MM-GBSA)_ (kcal/mol)
ACE2	–	− 87.9 ± 0.5	12.9 ± 0.6
Beta-27	–	− 100.8 ± 0.3	0.0 ± 0.4
Beta-27-D01	− 51.26	− 111.2 ± 0.4	− 10.4 ± 0.5
Beta-27-D02	− 49.80	− 100.1 ± 0.5	0.7 ± 0.6
Beta-27-D03	− 49.01	− 115.1 ± 0.4	− 14.3 ± 0.5
Beta-27-D04	− 47.66	− 98.5 ± 0.5	2.3 ± 0.6
Beta-27-D05	− 46.89	− 88.1 ± 0.5	12.7 ± 0.6
Beta-27-D06	− 46.81	− 109.3 ± 0.4	− 8.5 ± 0.5
Beta-27-D07	− 46.54	− 87.7 ± 0.4	13.1 ± 0.5
Beta-27-D08	− 46.45	− 98.7 ± 0.4	2.1 ± 0.5
Beta-27-D09	− 46.43	− 113.9 ± 0.5	− 13.1 ± 0.6
Beta-27-D10	− 46.26	− 120.7 ± 0.7	− 19.9 ± 0.8

In terms of binding free energy components (Figure S2), the van der Waals energy and non-polar solvation terms of Beta-27, Beta-27-D01, Beta-27-D03, Beta-27-D06, Beta-27-D09, and Beta-27-D10 Fabs have favorable contributions to the predicted binding affinities to Omicron RBD. The electrostatic interaction term of Beta-27 Fab has unfavorable contribution to the predicted binding affinity, while those of Beta-27-D01, Beta-27-D03, Beta-27-D06, Beta-27-D09, and Beta-27-D10 Fabs have favorable contribution to the predicted binding affinities. The polar solvation term of Beta-27 Fab has favorable contribution to the predicted binding affinity, while those of Beta-27-D01, Beta-27-D03, Beta-27-D06, Beta-27-D09, and Beta-27-D10 Fabs contribute unfavorably to the predicted binding affinities.

The redesigned Beta-27 Fab with the best ΔG_bind (MM-GBSA)_ is Beta-27-D10 Fab (-120.7 ± 0.7 kcal/mol) and its predicted binding affinity is better than that of Beta-27 Fab with the ΔΔG_bind (MM-GBSA)_ value of − 19.9 ± 0.8 kcal/mol. The favorable binding of Beta-27-D10 Fab is mostly caused by the increase in favorable electrostatic interaction term as well as the increase in favorable van der Waals energy and non-polar solvation terms, as compared to those of Beta-27 Fab, but the unfavorable polar solvation term of Beta-27-D10 Fab is worse than that of Beta-27 Fab. Beta-27-D01, Beta-27-D03, Beta-27-D06, and Beta-27-D09 Fabs also have the predicted binding affinities better than that of Beta-27 Fab. The main component contributing to the favorable predicted binding affinity of Beta-27-D01 Fab is the van der Waals energy term. Furthermore, the favorable electrostatic interaction and non-polar solvation terms of Beta-27-D01 Fab are better than those of Beta-27 Fab. The favorable binding affinities of Beta-27-D03, Beta-27-D06, and Beta-27-D09 Fabs are caused by the substantial increase in the favorable electrostatic interaction terms. Additionally, the favorable van der Waals energy and non-polar solvation terms of Beta-27-D03, Beta-27-D06, and Beta-27-D09 Fabs are also better than that of Beta-27 Fab. However, the unfavorable polar solvation terms of these five designed Beta-27 Fabs are worse than that of Beta-27 Fab.

### Identification of important binding residues

**Figure 2:**
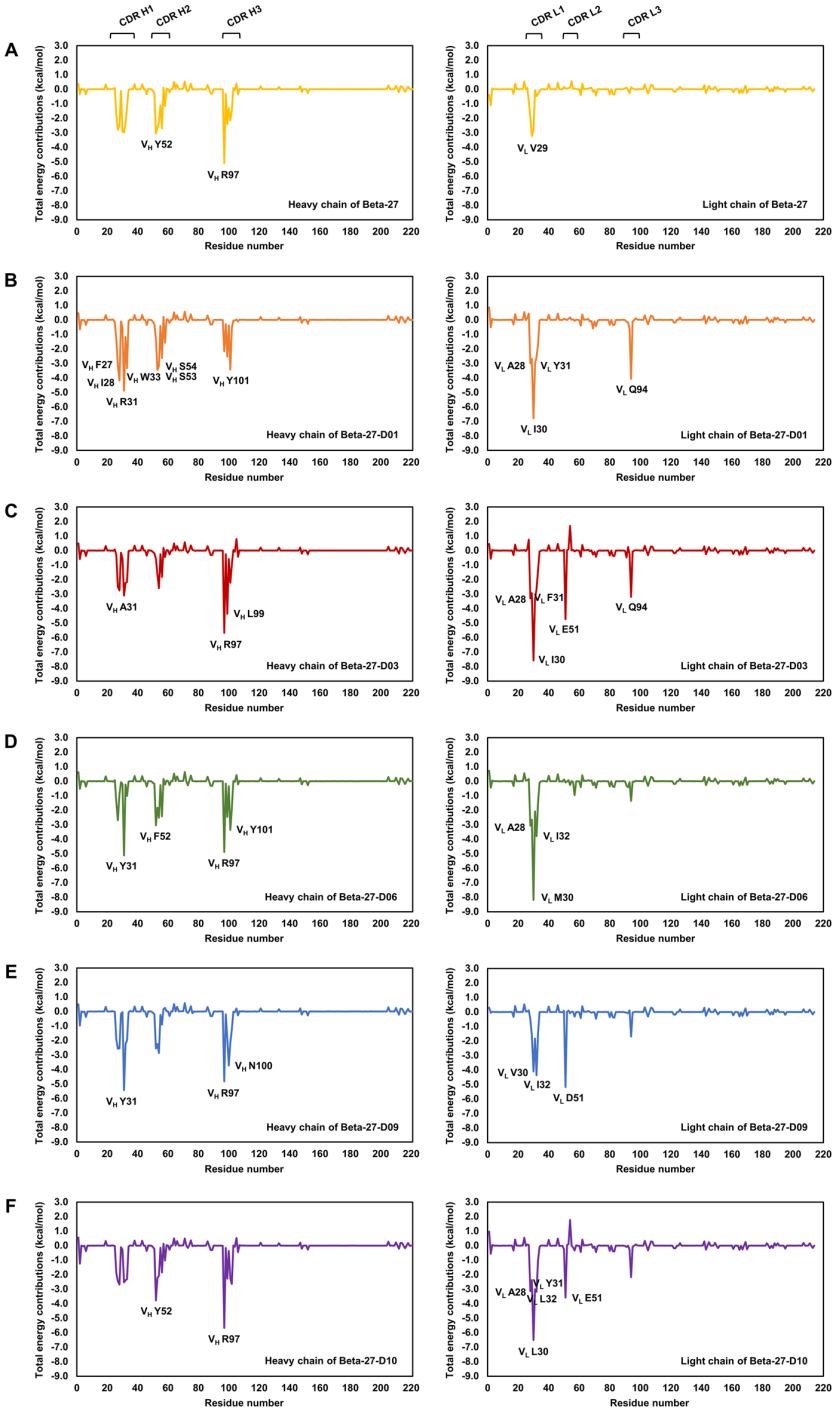
Per-residue free energy decomposition of (**A**) Beta-27, (**B**) Beta-27-D01, (**C**) Beta-27-D03, (**D**) Beta-27-D06, (**E**) Beta-27-D09, and (**F**) Beta-27-D10 Fabs binding to SARS-CoV-2-RBD Omicron variant. The left and right panels show per-residue free energy decomposition of residues in CDR H1, H2 and H3, and CDR L1, L2 and L3, respectively. Residues with high binding affinities that have the total energy contribution better than − 3.0 kcal/mol are labeled.

Per residue free energy decomposition was calculated to identify important binding residues of all systems to Omicron RBD (Fig. 2). An important binding residue was defined as a residue with the total energy contribution better than − 1.0 kcal/mol. Moreover, a residue with the total energy contribution better than − 3.0 kcal/mol was defined as a residue with high binding affinity.

The important binding residues of Beta-27 Fab are V_H_ residues G26, L27, T28, R30, S31, N32 and Y33 of H1, V_H_ residues Y52, S53, G54, G55, S56 and F58 of H2, V_H_ residues R97, L99, V100, V101 and Y102 of H3, I2 of the light chain, and V_L_ residues S28, V29 and S30 in L1. Furthermore, V_H_ Y52, V_H_ R97 and V_L_ V29 were predicted to have high binding affinities.

In terms of Beta-27-D01 Fab, the important binding residues are V_H_ residues G26, F27, I28, R31, T32 and W33 of H1, V_H_ residues S52, S53, S54, T56 and F58 of H2, V_H_ residues R97, L99 and Y101 of H3, V_L_ residues A28, V29, I30, Y31, L32 and F33 of L1, and V_L_ Q94 of L3. V_H_ residues F27, I28, R31 and W33 of H1, V_H_ residues S53 and S54 of H2, V_H_ Y101 of H3, V_L_ residues A28, I30 and Y31 of L1, and V_L_ Q94 of L3 were also predicted to be high binding affinity residues. The mutated residues including V_H_ residues F27, I28, R31, W33, S54 and Y101 as well as V_L_ residues A28, I30, Y31, L32, F33 and Q94 were predicted to have favorable increase of total energy contribution from − 2.8, − 2.5, − 3.0, − 1.2, − 2.2, − 2.1, − 1.9, − 2.8, − 0.1, − 0.5, − 0.3 and 0.1 kcal/mol in Beta-27 Fab to − 3.1, − 4.2, − 4.9, − 3.3, − 3.2, − 3.4, − 3.0, − 6.8, − 3.1, − 2.5, − 1.7 and − 4.1 kcal/mol in Beta-27-D01 Fab, respectively. Additionally, total energy contributions of other residues including V_H_ S53, V_H_ F58 and V_H_ L99 were favorably increased from − 2.7, − 1.2 and − 2.4 kcal/mol in Beta-27 Fab to − 3.5, − 1.6 and − 2.5 kcal/mol in Beta-27-D01 Fab, respectively.

For Beta-27-D03 Fab, V_H_ residues L27, N28, A31, N32 and W33 of H1, V_H_ residues S53, S54 and T56 of H2, V_H_ residues R97, L99 and V101 of H3, V_L_ residues A28, V29, I30, F31, L32 and Y33 of L1, V_L_ E51 of L2, and V_L_ Q94 of L3 were predicted to be important binding residues. V_H_ A31, V_H_ R97, V_H_ L99, V_L_ A28, V_L_ I30, V_L_ F31, V_L_ E51 and V_L_ Q94 were predicted to have high binding affinity. Additionally, total energy contributions of mutated residues including V_H_ residues N28, A31, W33 and S54, and V_L_ residues A28, I30, F31, L32, Y33, E51 and Q94 were favorably increased from − 2.5, − 3.0, − 1.2, − 2.2, − 1.9, − 2.8, − 0.1, − 0.5, − 0.3, 0.0 and 0.1 kcal/mol in Beta-27 Fab to − 2.7, − 3.1, − 2.2, − 2.6, − 3.3, − 7.6, − 3.3, − 2.7, − 1.4, − 4.7 and − 3.2 kcal/mol in Beta-27-D03 Fab, respectively. Moreover, total energy contributions of V_H_ residues R97, L99 and V101 of H3 were favorably increased from − 5.1, − 2.4 and − 2.1 kcal/mol in Beta-27 Fab to − 5.7, − 4.4 and − 2.2 kcal/mol in Beta-27-D03 Fab, respectively.

The important binding residues of Beta-27-D06 Fab are V_H_ residues G26, L27, Y31 and V33 of H1, V_H_ residues F52, S53, S54 and T56 of H2, V_H_ residues R97, L99, Y101 and L102 of H3, V_L_ residues A28, V29, M30, Q31, I32 and Y33 of L1, and V_L_ E94 of L3. Furthermore, V_L_ M30 (L1) was predicted to have the highest binding affinity, followed by V_H_ Y31 (H1), V_H_ R97 (H3), V_L_ I32 (L1), V_H_ Y101 (H3), V_L_ A28 (L1) and V_H_ F52 (H2), respectively. The mutated residues including V_H_ residues Y31, S54, Y101, and L102 as well as V_L_ residues A28, M30, Q31, I32, Y33, and E94 were predicted to have favorable increase in the total energy contribution from − 3.0, − 2.2, − 2.1, − 1.5, − 1.9, − 2.8, − 0.1, − 0.5, − 0.3 and 0.1 kcal/mol in Beta-27 Fab to − 5.1, − 2.5, − 3.4, − 1.9, − 3.1, − 8.2, − 2.1, − 3.8, − 1.5 and − 1.4 kcal/mol in Beta-27-D06 Fab, respectively. Additionally, the total energy contribution of V_H_ L99 was favorable increased from − 2.4 kcal/mol in Beta-27 Fab to − 2.5 kcal/mol in Beta-27-D06 Fab.

For Beta-27-D09 Fab, the important binding residues are V_H_ residues G26, L27, N28, Y31, N32 and W33 of H1, V_H_ residues Y52, S53 and S54 of H2, V_H_ residues R97, L99, N100, V101 and Y102 of H3, V_L_ residues I29, V30, F31, I32 and Y33 of L1, V_L_ D51 of L2 and V_L_ E94 of L3. V_H_ Y31 of H1, V_H_ residues R97 and N100 of H3, V_L_ residues V30 and I32 of L1 and V_L_ D51 of L2 were also predicted to be high binding affinity residues. Additionally, the total energy contributions of mutated residues including V_H_ residues N28, Y31, W33, S54 and N100, and V_L_ residues V30, F31, I32, Y33, D51 and E94 were favorably increased from − 2.5, − 3.0, − 1.2, − 2.2, − 1.3, − 2.8, − 0.1, − 0.5, − 0.3, 0.0 and 0.1 kcal/mol in Beta-27 Fab to − 2.6, − 5.4, − 2.0, − 2.9, − 3.7, − 4.1, − 1.8, − 4.4, − 1.5, − 5.2 and − 1.7 kcal/mol in Beta-27-D09 Fab, respectively. Furthermore, total energy contributions of other residues including V_H_ G26 and V_H_ V101 were favorably increased from − 1.8 and − 2.1 kcal/mol in Beta-27 Fab to − 1.9 and − 2.2 kcal/mol in Beta-27-D09 Fab, respectively.

In terms of Beta-27-D10 Fab, V2 (heavy chain), V_H_ residues G26, L27, V28, S31, N32 and W33 of H1, V_H_ residues Y52, S53, S54, T56 and F58 of H2, V_H_ residues R97, L99, Y101 and L102 of H3, V_L_ residues A28, I29, L30, Y31, L32 and I33 of L1, V_L_ E51 (L2) and V_L_ E94 (L3) were predicted to be important binding residues. Moreover, V_L_ L30 (L1) was predicted to have the highest binding affinity, followed by V_H_ R97 (H3), V_H_ Y52 (H2), V_L_ E51 (L2), V_L_ L32 (L1), V_L_ A28 (L1) and V_L_ Y31 (L1), respectively. Moreover, total energy contributions of mutated residues including V_H_ residues V28, W33, Y101 and L102 as well as V_L_ residues A28, L30, Y31, L32, I33, E51 and E94 were favorably increased from − 2.5, − 1.2, − 2.1, − 1.5, − 1.9, − 2.8, − 0.1, − 0.5, − 0.3, 0.0 and 0.1 kcal/mol in Beta-27 Fab to − 2.7, − 2.3, − 2.3, − 2.6, − 3.1, − 6.5, − 3.0, − 3.2, − 1.0, − 3.6 and − 2.2 kcal/mol in Beta-27-D10 Fab, respectively. Furthermore, the total energy contributions of V2, V_H_ G26, V_H_ N32, V_H_ Y52 and V_H_ R97 of the heavy chain were also favorably increased from − 0.3, − 1.8, − 2.3, − 3.1 and − 5.1 kcal/mol in Beta-27 Fab to − 1.2, − 1.9, − 2.4, − 3.8 and − 5.7 kcal/mol in Beta-27-D10 Fab, respectively.

### Hydrogen bond (H-bond) and pi interactions

**Figure 3:**
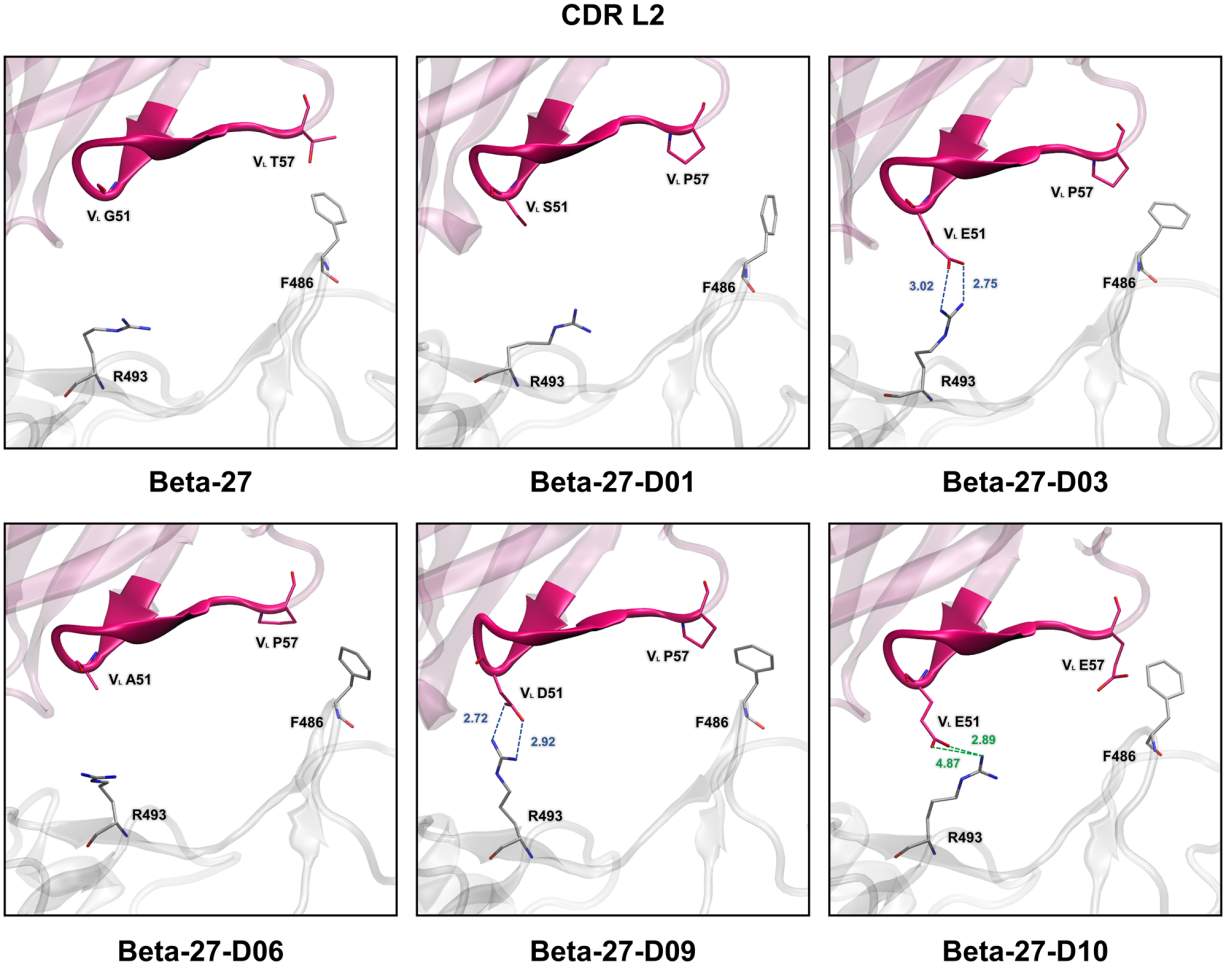
Key binding interactions between Omicron RBD and CDR L2 of the light chain of Beta-27, Beta-27-D01, Beta-27-D03, Beta-27-D06, Beta-27-D09, and Beta-27-D10 Fabs. Strong and medium H-bonds are shown in blue and green dashed lines, respectively. Distance (Å) of strong and medium H-bonds are labeled in blue and green, respectively.

**Figure 4:**
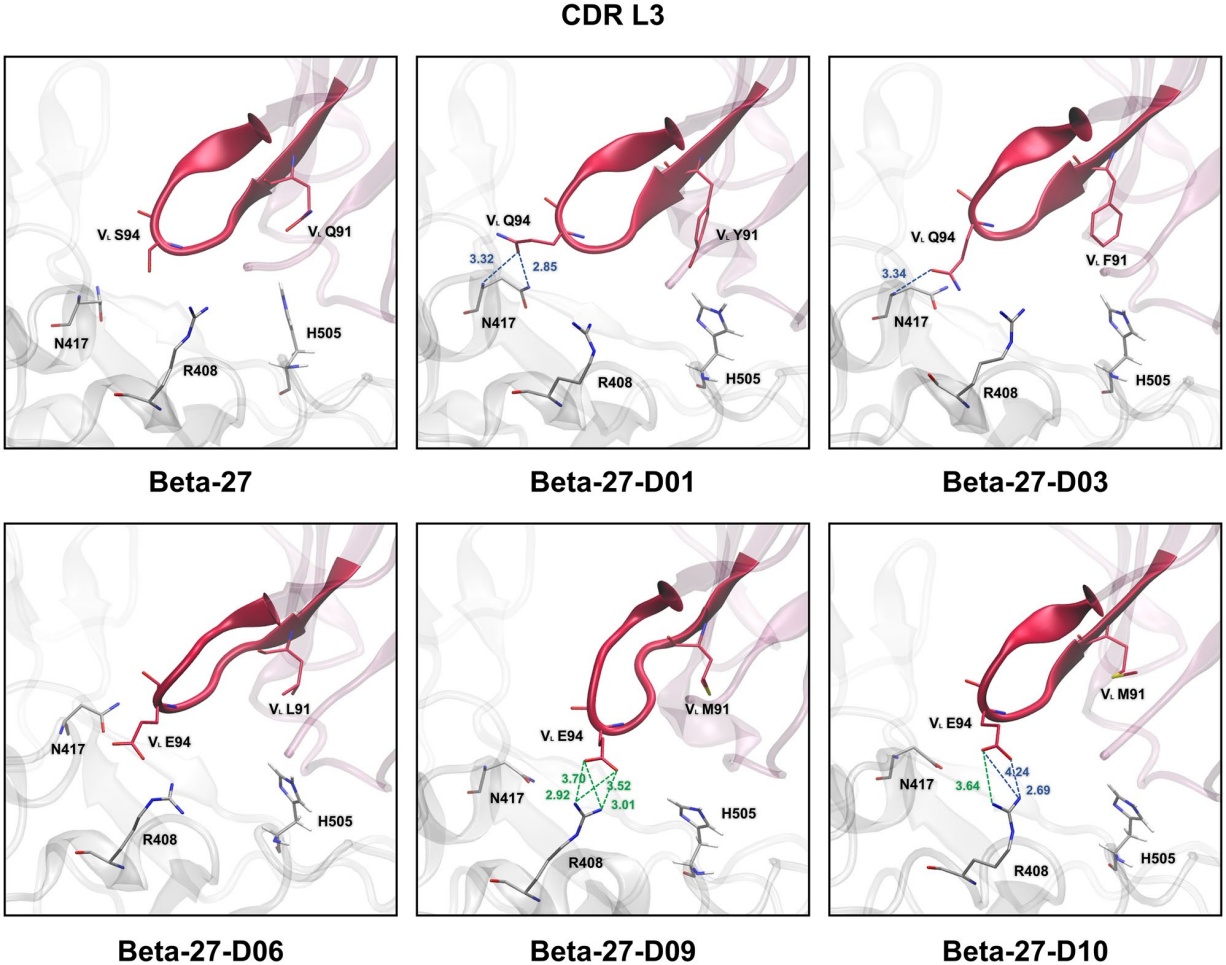
Key binding interactions between SARS-CoV-2-RBD Omicron variant and CDR L3 of the light chain of Beta-27, Beta-27-D01, Beta-27-D03, Beta-27-D06, Beta-27-D09, and Beta-27-D10 Fabs. Strong and medium H-bonds are shown in blue and green dashed lines, respectively. Distance (Å) of strong and medium H-bonds are labeled in blue and green, respectively.

To identify important H-bonds and pi interactions, H-bond occupations (Tables 3 and S1-S6) and the numbers of pi-pi, cation-pi, anion-pi, sigma-pi, and alkyl-pi interactions (Tables 4 and S7) were analyzed. The key binding interactions of Beta-27 Fab and best five designed Beta-27 Fab to SARS-CoV-2-RBD Omicron variant are shown in Figs. 3, 4 and S3-S6.

**Table 3:** Numbers of H-bonds of Beta-27 Fab, Beta-27-D01 Fab, Beta-27-D03 Fab, Beta-27-D06 Fab, Beta-27-D09 Fab, and Beta-27-D10 Fab involved in SARS-CoV-2-RBD Omicron variant binding (*s* strong H-bond, *m* medium H-bond, *w* weak H-bond, and *vw* very weak H-bond).

System	Number of H-bonds					Residue that forms a H-bond with SARS-CoV-2-RBD Omicron variant						
	Strong	Medium	Weak	Very weak	Total	Outside CDRs	Heavy chain			Light chain		
							CDR H1	CDR H2	CDR H3	CDR L1	CDR L2	CDR L3
Beta-27	15	2	8	9	34	D1(L) (w)	G26 (s,vw)	Y52 (vw)	R97 (s)	Q27 (m)	–	–
							T28 (s)	S53 (s)	Y102 (vw)	S28 (s)		
							R30 (s)	G54 (w)		S30 (s,vw)		
							S31 (s,vw)	G55 (w,vw)		S32 (vw)		
							N32 (s)	S56 (s,m,w,vw)				
							Y33 (s,vw)					
Beta-27-D01	11	2	6	11	30	D1(L) (vw)	S25 (vw)	S53 (s,w)	R97 (m,vw)	E27 (w,vw)	–	Q94 (s)
						G67(L) (vw)	G26 (w,vw)	S54 (s,m)		A28 (s)		
							I28 (s)	T56 (s)		I30 (vw)		
							R31 (s,w)			Y31 (w,vw)		
							W33 (s)					
Beta-27-D03	13	3	7	13	36	D1(L) (vw)	G26 (vw)	S52 (m)	R97 (s)	E27 (w,vw)	E51 (s,w)	Q94 (s,w,vw)
							N28 (s,w,vw)	S53 (s,vw)		A28 (s)		
							A31 (s)	S54 (s,m,vw)		I30 (m)		
							N32 (s)	T56 (s,vw)		Y33 (s)		
Beta-27-D06	7	2	8	20	37	–	G26 (m)	S53 (s,w,vw)	R97 (s,vw)	A28 (s)	Y54 (vw)	Y92 (vw)
							L27 (vw)	S54 (s,vw)	Y101 (w)	M30 (s)		E94 (w,vw)
							D28 (w)	T56 (s)		Q31 (m,vw)		
							Y31 (w,vw)	Y58 (vw)		Y33 (w,vw)		
Beta-27-D09	11	8	4	14	37	E1(H) (vw) N76(H) (vw)	G26 (s,vw)	S53 (s,m,vw)	R97 (s,vw)	I32 (m)	D51 (s,w)	E94 (m)
							L27 (vw)	S54 (s,w,vw)	N100 (s)	Y33 (vw)		
							N28 (s,vw)	T56 (m,w)				
							Y31 (s)					
							N32 (s)					
							W33 (m)					
Beta-27-D10	8	11	9	20	48	E1(H) (vw)	G26 (m,vw)	Y52 (vw)	R97 (s,vw)	E27 (w,vw)	E51 (m,w)	E94 (s,m,w)
							V28 (m)	S53 (s,m,w,vw)	Y101 (m,vw)	A28 (s)		
							S31 (s)	S54 (m,w,vw)	D105 (vw)	L30 (vw)		
							N32 (s)	T56 (m,w,vw)		Y31 (m)

**Table 4:** Numbers of pi interactions of Beta-27 Fab, Beta-27-D01 Fab, Beta-27-D03 Fab, Beta-27-D06 Fab, Beta-27-D09 Fab, and Beta-27-D10 Fab involved in SARS-CoV-2-RBD Omicron variant binding.

System	Number of pi interactions					
	Pi-pi	Cation-pi	Anion-pi	Sigma-pi	Alkyl-pi	Total
Beta-27	3	1	1	–	2	7
Beta-27-D01	3	6	–	1	11	21
Beta-27-D03	2	3	–	1	8	14
Beta-27-D06	3	5	–	4	12	24
Beta-27-D09	3	5	–	1	8	17
Beta-27-D10	5	3	–	2	6	16

For H1, H2 and H3 of Beta-27 Fab, V_H_ residues G26, S31 and Y33 of H1 were predicted to form strong H-bonds with N477, Y473 and L455 of Omicron RBD, respectively. V_H_ T28 and V_H_ N32 were predicted to form two strong H-bonds with the backbone carbonyl of A475 of Omicron RBD. Two strong H-bonds were also predicted to form between V_H_ R30 and K458 of Omicron RBD. Moreover, there is one pi–pi interaction between V_H_ Y33 and F456 of Omicron RBD. V_H_ S53 of CDR H2 was predicted to form two strong H-bonds with R457 of Omicron RBD. V_H_ S56 was also predicted to form strong and medium H-bonds with D420 and Y421 of Omicron RBD, respectively. Additionally, V_H_ Y52 was predicted to form one pi–pi interaction with Y421 of Omicron RBD. Two strong H-bonds were predicted to form between V_H_ R97 of H3 with N487 of Omicron RBD. Furthermore, there are one pi-pi, one cation-pi and one alkyl-pi interactions formed between H3 and Omicron RBD. In terms of L1, L2 and L3, V_L_ residues Q27 and S30 of L1 were predicted to form medium and strong H-bonds with G504 and H505 of Omicron RBD, respectively. V_L_ S28 was also predicted to form two strong H-bonds with T500 and G502 of Omicron RBD. One alkyl-pi interaction was predicted to form between V_L_ V29 and H505 of Omicron RBD. Moreover, E1 in the heavy chain was predicted to form one anion-pi with F486 of Omicron RBD.

In terms of H1, H2 and H3 of Beta-27-D01 Fab, backbones of mutated residues V_H_ I28, V_H_ R31 and V_H_ W33 of H1 were predicted to form three strong H-bonds with A475, Y473 and L455 of Omicron RBD, respectively. There are one pi-pi, one cation-pi and three alkyl-pi interactions formed between these mutated residues (H1) and Omicron RBD. V_H_ S53 (H2) was predicted to form two strong H-bonds with R457 of Omicron RBD. The mutated residue V_H_ S54 was predicted to form two strong H-bonds with Y421 and N460 of Omicron RBD. There is one predicted medium H-bond formed between the mutated residue V_H_ S54 and Y421 of Omicron RBD. One strong H-bond was also predicted to form between the mutated residue V_H_ T56 and D420 of Omicron RBD. Additionally, V_H_ R97 (H3) was predicted to form a medium H-bond with N487 of Omicron RBD. There are two predicted cation-pi interactions between V_H_ R97 and F486 of Omicron RBD. V_H_ L99 was predicted to form two alkyl-pi interactions with F456 and Y489 of Omicron RBD. Moreover, the mutated residue V_H_ Y101 (H3) was predicted to form two cation-pi and one alkyl-pi interactions with R493 of Omicron RBD. For L1, L2 and L3 of Beta-27-D01 Fab, one strong H-bond was predicted to form between the mutated residue V_L_ A28 (L1) and G502 of Omicron RBD. Furthermore, V_L_ V29 was predicted to form sigma-pi and alkyl-pi interactions with H505 of Omicron RBD. There are one pi-pi, one cation-pi and three alkyl-pi interactions formed between these mutated residues (L1) and Omicron RBD. The mutated residue V_L_ Q94 (L3) was predicted to form two strong H-bonds with N417 of Omicron RBD. One pi–pi interaction was predicted to form between the mutated residue V_L_ Y91 and H505 of Omicron RBD. Moreover, there is one alkyl-pi interaction formed between V2 in the heavy chain of Beta-27-D01 Fab and F486 of Omicron RBD.

For H1, H2 and H3 of Beta-27-D03 Fab, V_H_ N32 and the mutated residue V_H_ N28 (H1) were predicted to form two strong H-bonds with the backbone carbonyl of A475 of Omicron RBD. The mutated residue V_H_ A31 was predicted to form one strong H-bond and one alkyl-pi interaction with Y473 of Omicron RBD. Furthermore, there is one pi–pi interaction formed between the mutated V_H_ residue W33 and Y421 of Omicron RBD. For H2, one strong H-bond was predicted to form between V_H_ S53 and R457 of Omicron RBD. The backbone of the mutated residue V_H_ S54 was additionally predicted to form strong and medium H-bonds with D420 and Y421 of Omicron RBD, respectively. There is a strong H-bond formed between the mutated residue V_H_ T56 and D420 of Omicron RBD. Moreover, the mutated residue V_H_ S52 was predicted to form a medium H-bond with Y421 of Omicron RBD. V_H_ R97 (H3) was predicted to form two strong H-bonds and one cation-pi interaction with N487 and F486 of Omicron RBD, respectively. Additionally, three alkyl-pi interactions were predicted to form between V_H_ L99 (H3) and F456, Y473 and Y489 of Omicron RBD. In terms of L1, L2 and L3 of Beta-27-D03 Fab, the mutated residues V_L_ A28 and V_L_ Y33 of L1 were predicted to form two strong H-bonds with G502 and Y453 of Omicron RBD, respectively. The mutated residue V_L_ I30 was also predicted to form one medium H-bond with H505 of Omicron RBD. One sigma-pi and one alkyl-pi interactions were predicted to form between V_L_ V29 and H505 of Omicron RBD. Additionally, there are one pi-pi, two cation-pi and two alkyl-pi interactions formed between these mutated residues (L1) and Omicron RBD. The mutated residue V_L_ E51 (L2) was predicted to form two strong H-bonds with R493 of Omicron RBD. Moreover, strong H-bonds were predicted to form between the mutated residue V_L_ Q94 (L3) and N417 of Omicron RBD. Additionally, V2 in the heavy chain was predicted to form one alkyl-pi interaction with F486 of Omicron RBD.

For H1, H2 and H3 of Beta-27-D06 Fab, V_H_ G26 (H1) was predicted to form a medium H-bond with N477 of Omicron RBD. There are one pi-pi, one cation-pi, one sigma-pi and three alkyl-pi interactions formed between H1 and Omicron RBD. A strong H-bond was predicted to form between V_H_ S53 (H2) and R457 of Omicron RBD. The mutated residues V_H_ S54 and V_H_ T56 were predicted to form two strong H-bonds with D420 of Omicron RBD. The mutated residue V_H_ F52 was also predicted to form one pi–pi interaction with Y421 of Omicron RBD. For H3, V_H_ R97 was predicted to form two strong H-bonds and one cation-pi interaction with N487 and F486 of Omicron RBD, respectively. Moreover, one cation-pi, one sigma-pi and five alkyl-pi interactions were predicted to form between H3 and Omicron RBD. In terms of L1, L2 and L3 of Beta-27-D06 Fab, two strong H-bonds were predicted to form between the mutated residues V_L_ A28 and V_L_ M30 of L1 and G502 and H505 of Omicron RBD, respectively. The mutated residue V_L_ Q31 was predicted to form a medium H-bond with Y501 of Omicron RBD. One sigma-pi and one alkyl-pi interactions were predicted to form between V_L_ V29 and H505 of Omicron RBD. Additionally, other residues of L1 including the mutated residues V_L_ M30 and V_L_ Y33 were predicted to form one pi-pi, two cation-pi, one sigma-pi and two alkyl-pi interactions with Omicron RBD. Although L2 was not predicted to form any strong or medium H-bonds, the mutated residue V_L_ P57 (L2) was predicted to form an alkyl-pi interaction with F486 of Omicron RBD.

In terms of H1, H2 and H3 of Beta-27-D09 Fab, V_H_ residues G26 and N32 of H1 were predicted to form two strong H-bonds with N477 and A475 of Omicron RBD, respectively. The backbones of the mutated residues V_H_ N28 and V_H_ Y31 were predicted to form two strong H-bonds with A475 and Y473 of Omicron RBD, respectively. There are one predicted medium H-bond and one predicted pi-pi interaction formed between the mutated residue V_H_ W33 and Y421 of Omicron RBD. Moreover, one cation-pi and alkyl-pi interactions were predicted to form between the mutated residue V_H_ Y31 and K458 of Omicron RBD. V_H_ S53 (H2) was predicted to form strong and medium H-bonds with R457 and Y421 of Omicron RBD, respectively. The mutated residues V_H_ S54 and V_H_ T56 were predicted to form strong and medium H-bonds with D420 and T415 of Omicron RBD, respectively. Additionally, there is one pi–pi interaction formed between V_H_ Y52 and Y421 of Omicron RBD. For H3, V_H_ R97 was predicted to form two strong H-bonds and one cation-pi interaction with N487 and F486 of Omicron RBD, respectively. The mutated residue V_H_ N100 was predicted to form a strong H-bond with L455 (backbone) of Omicron RBD. Other residues of H3 including V_H_ residues L99 and Y102 were predicted to form two alkyl-pi interactions with Omicron RBD. For L1, L2 and L3, one medium H-bond was predicted to form between the mutated residue V_L_ I32 (L1) and R493 of Omicron RBD. Furthermore, there are one pi-pi, three cation-pi, one sigma-pi and three alkyl-pi interactions formed between these mutated residues (L1) and Omicron RBD. For L2, the mutated residue V_L_ D51 was predicted to form two strong H-bonds with the backbone carbonyl of R493 of Omicron RBD. Additionally, there is one alkyl-pi interaction formed between the mutated residue V_L_ P57 and F486 of Omicron RBD. The mutated residue V_L_ E94 (L3) was predicted to form four medium H-bonds with R408 of Omicron RBD. Furthermore, one alkyl-pi interaction was predicted to form between V2 of the heavy chain and F486 of Omicron RBD.

For H1, H2 and H3 of Beta-27-D10 Fab, V_H_ residues S31 and N32 of H1 were predicted to form two strong H-bonds with Y473 and A475 of Omicron RBD, respectively. Other residues of H1 including V_H_ G26 and the mutated residue V_H_ V28 were predicted to form two medium H-bonds with N477 and A475 of Omicron RBD, respectively. Additionally, there are two pi-pi interactions formed between the mutated residue V_H_ W33 (H1) and Omicron RBD. V_H_ S53 (H2) was predicted to form strong and medium H-bonds with R457 and Y421 of Omicron RBD, respectively. The mutated residues V_H_ S54 and V_H_ T56 were predicted to form two medium H-bonds with Y421 and D420 of Omicron RBD, respectively. Moreover, there is one predicted pi–pi interaction formed between V_H_ Y52 and Y421 of Omicron RBD. For H3, two strong H-bonds and one cation-pi interaction were predicted to form between the mutated residue V_H_ R97 and N487 and F486 of Omicron RBD, respectively. The mutated residue V_H_ Y101 was predicted to form two medium H-bonds with R403 and E406 of Omicron RBD. Furthermore, there are one pi-pi, one cation-pi and two alkyl-pi interactions between H3 and Omicron RBD. In terms of L1, L2 and L3 of Beta-27-D10 Fab, the mutated residues V_L_ A28 and V_L_ Y31 of L1 were predicted to form strong and medium H-bonds with G502 and Y501 of Omicron RBD, respectively. Moreover, there are one pi-pi, one cation-pi, two sigma-pi and three alkyl-pi interactions formed between these mutated residues (L1) and Omicron RBD. For L2, two medium H-bonds were predicted to form between the mutated residue V_L_ E51 and R493 of Omicron RBD. Additionally, there are two strong and one medium H-bonds formed between the mutated residue V_L_ E94 (L3) and R408 of Omicron RBD. Moreover, V2 in the heavy chain was additionally predicted to form one alkyl-pi interaction with F486 of Omicron RBD.

## Discussion

The COVID-19 pandemic, caused by SARS-CoV-2, is responsible for large numbers of cases and deaths worldwide. SARS-CoV-2-RBD initially binds to ACE2-PD to enter human cells. Blocking binding interactions between SARS-CoV-2-RBD and ACE2-PD using antibodies is an effective therapeutic solution for COVID-19. Example of neutralizing antibody that the U.S. Food and Drug Administration has authorized for current use during an emergency to treat COVID-19 patients is ACTEMRA (tocilizumab)^43^.

The previous experimental study discovered that Beta-27 Fab, which was obtained by digesting the full IgG antibodies that were isolated from a patient infected with SARS-CoV-2 Beta variant, can neutralize Victoria and the previous VOCs such as Alpha, Beta, Gamma, and Delta^44^. Omicron variant has emerged as VOC of COVID-19^10^. Since Beta-27 Fab can neutralize various variants, we hypothesized that it might be able to neutralize the Omicron variant as well. However, at the time that this study was initially started, the knowledge on the binding between Beta-27 Fab and Omicron RBD is limited. Moreover, there was no crystal structure of the Omicron RBD or Omicron subvariant RBD binding to Fab at that time. However, there was a crystal structure of Beta-27 Fab/Beta RBD complex (PDB code: 7PS1^44^) available in the protein data bank, and Beta-27 was reported to neutralize various VOCs. Furthermore, the sequence alignment between Beta RBD and Omicron RBD (Figure S7) show that the majority of the residues involved in binding between Beta RBD and Beta-27 are different from additionally mutated residues in Omicron RBD. Therefore, using the crystal structure of the Beta RBD binding to Beta-27 Fab as a template for designing Fab that can potentially bind to the Omicron RBD seemed to be a reasonable approach at the time that our study was initially conducted.

To investigate and increase the binding affinity of Beta-27 Fab to Omicron RBD, we employed RAbD and MD to redesign all CDRs of Beta-27 Fab so that their predicted binding affinities to Omicron RBD are better than those of ACE2 and Beta-27 Fab. After computational design, the redesigned Beta-27 Fabs with the top ten best ΔG_bind (Rosetta)_ were selected for MD to compute their predicted binding affinities by the MM-GBSA method (ΔG_bind (MM-GBSA)_). Five redesigned Beta-27 Fabs (Beta-27-D01, Beta-27-D03, Beta-27-D06, Beta-27-D09, and Beta-27-D10 Fabs) were predicted to bind to Omicron RBD better than ACE2 and Beta-27 Fab, suggesting that they should be able to experimentally bind to Omicron RBD better than Beta-27 Fab and ACE2. Furthermore, the predicted binding affinity of ACE2/Omicron RBD complex (ΔG_bind (MM-GBSA)_ =  − 87.9 ± 0.5 kcal/mol) is better than that of ACE2/SARS-CoV-2-RBD complex (ΔG_bind (MM-GBSA)_ =  − 71.2 ± 0.4 kcal/mol)^45^, supporting the experimental result that ACE2 bind to Omicron RBD (*K*_*D*_ = 38.9 ± 10.5 nM^21^) better than the wild type (*K*_*D*_ = 75.5 ± 2.1 nM^21^). The ranking of predicted binding affinities of Beta-27 Fab, ACE2 and all designed Beta-27 Fabs to Omicron RBD from best to worst is Beta-27-D10 Fab > Beta-27-D03 Fab > Beta-27-D09 Fab > Beta-27-D01 Fab > Beta-27-D06 Fab > Beta-27 Fab > ACE2. Additionally, Beta-27 Fab binds to Omicron RBD at a binding site similar to ACE2, and the binding poses of Beta-27 Fab and five best designed Beta-27 Fabs to Omicron RBD are relatively similar, suggesting that Beta-27 Fab and designed Beta-27 Fabs could potentially block ACE2 and Omicron RBD binding.

The most promising designed Beta-27 Fab is Beta-27-D10 Fab because of its highest predicted binding affinity to Omicron RBD, which is substantially better than ACE2 (about 33 kcal/mol) and Beta-27 Fab (about 20 kcal/mol). This finding is supported by the fact that its total number of predicted H-bonds is substantially higher than those of other systems. Its total number of pi interactions is also higher than those of Beta-27 Fab and Beta-27-D03 Fab. However, the total number of pi interactions of Beta-27-D10 Fab is lower than those of Beta-27-D01 Fab, Beta-27-D06 Fab and Beta-27-D09 Fab. Although the predicted number of strong H-bonds of Beta-27-D10 Fab is lower than those of Beta-27 Fab, Beta-27-D01 Fab, Beta-27-D03 Fab, and Beta-27-D09 Fab, it has the highest number of medium H-bonds. Its total numbers of weak and very weak H-bonds are also more than other systems. The results from per-residue free energy decomposition suggest V2 (heavy chain), V_H_ residues G26, L27, V28, S31, N32 and W33 of H1, V_H_ residues Y52, S53, S54, T56 and F58 of H2, V_H_ residues R97, L99, Y101 and L102 of H3, V_L_ residues A28, I29, L30, Y31, L32 and I33 of L1, V_L_ E51 of L2 and V_L_ E94 of L3 as important binding residues. Moreover, Beta-27-D10 Fab was predicted to cause substantial favorable increase in the total energy contributions of the mutated residues including V_H_ residues V28, W33, Y101 and L102, and V_L_ residues A28, L30, Y31, L32, I33, E51 and E94, and other residues including V2, V_H_ G26, V_H_ N32, V_H_ Y52 and V_H_ R97 of the heavy chain as compared to those of Beta-27 Fab. Overall, the enhanced binding affinity between Beta-27-D10 Fab and Omicron RBD is mostly caused by the increase in the binding interactions of the light chain as compared to those of Beta-27 Fab. This finding is supported by the fact that the total numbers of H-bonds and pi interactions of the light chain of Beta-27-D10 Fab are higher than those of Beta-27 Fab. Additionally, L2 and L3 of Beta-27-D10 form H-bonds with Omicron RBD, while those of Beta-27 Fab do not form any H-bonds with Omicron RBD. In terms of the binding interactions between the heavy chain and Omicron RBD, although the total number of predicted strong H-bonds of Beta-27-D10 Fab is lower than that of Beta-27 Fab, its total numbers of predicted medium H-bonds and pi interactions are higher than those of Beta-27 Fab.

As shown in Figure S8, the binding pose to Omicron RBD of the most promising redesigned Beta-27 Fab (Beta-27-D10 Fab) is different from those of P2G3 Fab (PDB code: 7QTK)^55^ and S3H3 (PDB code: 7WK9 and 7WKA)^56^, which were determined by electron microscopy, probably because the sequence of Beta-27 Fab is very different from those of P2G3 (Figure S9). Recently, Beta-27 has been experimentally proven with the crystal structures that Beta-27 can bind to Omicron BA.4/5 RBD (PDB code: 7ZXU^57^) and Omicron BA.2.12.1 RBD (PDB code: 8BH5^58^) with similar binding pose to Beta RBD (PDB code: 7PS1^44^), suggesting that Beta-27 Fab should be able to bind to Omicron RBD and also supporting that Beta-27 Fab may be a good template for designing Fab that can bind Omicron RBD. Additionally, the binding pose of Beta-27-D10 Fab to Omicron RBD is very similar to those of Beta-27 Fabs binding to Omicron BA.4/5 RBD (PDB code: 7ZXU)^57^ and Omicron BA.2.12.1 RBD (PDB code: 8BH5)^58^ with the backbone RMSD values of 1.42 Å and 1.35 Å, respectively (these two crystal structures were determined by x-ray crystallography and released in the protein data bank after we already performed computational protein design and molecular dynamics simulations).

In terms of binding interactions between CDRs of the heavy chain and Omicron RBD, H1, H2 and H3 of Beta-27 Fab and the five best designed Beta-27 Fabs were predicted to form favorable binding interactions with Omicron RBD. For binding interactions between CDRs of the light chain and Omicron RBD, Beta-27 Fabs and the five best designed Beta-27 Fabs were all predicted to form favorable binding interactions between L1 and Omicron RBD. L2 and L3 of Beta-27-D03, Beta-27-D06, Beta-27-D09 and Beta-27-D10 Fabs form favorable binding interactions to Omicron RBD, while those of Beta-27 Fab do not. In addition to L1, L3 of Beta-27-D01 Fab was predicted to form favorable binding interactions to Omicron RBD. Our results suggest that the enhanced binding interactions of the light chain, especially CDR L2 and L3, are most likely responsible for the increased binding affinities of the five best designed Beta-27 Fabs as compared to those of Beta-27 Fab. These findings suggest L2 and L3 as promising design targets of Beta-27 Fab to further increase its binding affinity.

Using Beta-27 Fab as a template, we employed computational protein design and MD to design promising Fabs with better predicted binding affinities to Omicron RBD than human ACE2 receptor and Beta-27 Fab. The best five designed Beta-27 Fabs (Beta-27-D01, Beta-27-D03, Beta-27-D06, Beta-27-D09 and Beta-27-D10 Fabs) were predicted to bind better to Omicron RBD, as computed by the MM-GBSA method, than ACE2 and Beta-27 Fab. Beta-27-D10 Fab is the most promising designed Beta-27 Fab with substantially better predicted binding affinities to Omicron RBD than human ACE2 receptor (about 33 kcal/mol) and Beta-27 Fab (about 20 kcal/mol). The enhanced binding affinities of Beta-27-D01, Beta-27-D03, Beta-27-D06, Beta-27-D09 and Beta-27-D10 Fab are mostly caused by the increased binding interactions of the light chain (CDR L2 and L3). These results support CDR L2 and L3 as promising design targets to further increase the binding affinity of Beta-27 Fab. Beta-27-D01, Beta-27-D03, Beta-27-D06, Beta-27-D09 and Beta-27-D10 Fab are promising candidates that could potentially be used to disrupt the binding between ACE2 and Omicron RBD. In any case, experimental studies such as Omicron RBD-ACE2 binding inhibition and plaque reduction can be further conducted to confirm that they have virus neutralizing activity.

## Methods

### Structure preparation

The complex structure of ACE2 bound to SARS-CoV-2-RBD Omicron variant was obtained from the protein data bank (PDB code: 7TN0)^59^**.** Since there was no crystal structure of Beta-27 Fab bound to SARS-CoV-2-RBD Omicron variant available at the time that we started this study, the structure of Beta-27 Fab/SARS-CoV-2-RBD Omicron variant complex was constructed by modifying the crystal structure of Beta-27 Fab bound to SARS-CoV-2-RBD Beta variant (PDB code: 7PS1)^44^, using the LEaP module of AMBER18^60^. H +  + server^61^ was employed to protonate all ionizable amino acids at the physiological pH 7.4. The LEaP module was subsequently used to construct the final structure of the complex.

### Computational protein design

The structure of Beta-27 Fab/SARS-CoV-2-RBD Omicron variant complex was used as a design template. To increase the binding affinity between Beta-27 Fab and SARS-CoV-2-RBD Omicron variant, RosettaAntibodyDesign (RAbD)^51^ in RosettaDesign module of Rosetta3.12^62^ was employed to design the CDR H1, H2 and H3 of the heavy chain and CDR L1, L2 and L3 of the light chain of Beta-27 Fab. For CDR structural classifications of CDR H1, H2, H3, L1, L2 and L3, RAbD uses the Rosetta Antibody Design Database that can be obtained from PyIgClassify (http://dunbrack2.fccc.edu/pyigclassify). The RAbD protocol consists of outer and inner Monte Carlo cycles. In the inner cycle, each CDR residue was allowed to be any of standard amino acids using SequenceDesign (SeqDesign), and their structures were energetically minimized. 500 independent runs were performed, and the total of 500 conformations of designed sequences were obtained. The binding free energy (ΔG_bind (Rosetta)_) of each designed conformation was calculated in Rosetta Energy Unit (REU). The designed sequences/conformations with ΔG_bind (Rosetta)_ < 0 REU and are in the top ten best ΔG_bind (Rosetta)_ values were chosen for MD simulations.

### MD simulations and analyses

Using protein ff14SB^63^ and GLYCAM06j-1^64^ force field parameters, the LEaP module of AMBER18^60^ was employed to solvate the complexes of ACE2/SARS-CoV-2-RBD Omicron variant, Beta-27 Fab/SARS-CoV-2-RBD Omicron variant and designed Beta-27 Fabs/SARS-CoV-2-RBD Omicron variant in isomeric truncated octahedral TIP3P water boxes with the buffer distance of 13 Å. Then, the five steps minimization procedure was applied each system to reduced unfavorable interactions of complexes^45–47,65–77^. All steps include 2,500 steps of steepest-descent and 2,500 steps of conjugated gradient with different restrains on proteins. In the first step, the heavy atoms of protein were restrained with a force constant of 10 kcal/(mol Å), while the hydrogen atoms and water molecules were minimized. The force constants of 10, 5 and 1 kcal/(mol Å) were subsequently applied to restrain the backbone of protein in the second, third and fourth steps of minimizations, respectively. For the last step, the whole system was minimized with no restrain.

After minimization, all systems were simulated under the periodic boundary condition, using the GPU (CUDA) version of PMEMD module^78–80^. The SHAKE algorithm^81^ was employed to constrain all bonds relating to hydrogen atoms, allowing simulations with the time step of 0.002 ps. To control the simulation temperature, the Langevin dynamic technique^82^ was used with a collision frequency of 1 ps^−1^. All systems were heated from 0 to 310 K (physiological temperature) for 200 ps in the NVT ensemble, while the protein backbones were restrained with the force constant of 10 kcal/(mol Å). All systems were then equilibrated at 310 K for 300 ps in the NVT ensemble with no restraint. Finally, all systems were simulated in the NPT ensemble at 310 K and 1 atm for 100 ns.

In terms of analyses, the root mean square deviation (RMSD) values were calculated to elucidate the stability of each system. The last 20 ns trajectories of all systems with stable RMSD values were selected for further analyses. The molecular mechanics–generalized born surface area (MM-GBSA) method^52–54^ was employed to calculate the total binding free energies (ΔG_bind (MM-GBSA)_) of all systems to predict the binding affinities between ACE2/ Beta-27 Fab/ designed Beta-27 Fabs and SARS-CoV-2-RBD Omicron variant. The designed Beta-27 Fabs with better predicted binding affinities than Beta-27 Fab were selected for analyses in terms of decomposition of free energy per residue and binding interactions. Decomposition of free energy per residue was computed to identify important binding residues between Beta-27 Fab/ designed Beta-27 Fabs and SARS-CoV-2-RBD Omicron variant. For important binding interactions, H-bond and Pi interactions were analyzed. A H-bond was considered to occur with the following conditions: a proton donor − acceptor distance ≤ 3.5 Å and a donor-H-acceptor bond angle ≥ 120°^67–69,72^. H-bond was classified into four levels: (1) strong H-bonds (H-bond > 75%), (2) medium H-bonds (75% ≥ H-bond > 50%), (3) weak H-bonds (50% ≥ H-bond > 25%) and (4) very weak H-bonds (25% ≥ H-bond > 5%)^67,69–72^.

### Supplementary Information

Supplementary Information.

## Data Availability

All data generated or analysed during this study are included in this published article and its supplementary information files.
