# Supplementary material for: Computational redesign of Beta-27 Fab with substantially better predicted binding affinity to the SARS-CoV-2 Omicron variant than human ACE2 receptor
Source: Sci Rep. 2023 Sep 19;13:15476. doi: 10.1038/s41598-023-42442-1 (PMC10509195; doi:10.1038/s41598-023-42442-1)
Supplement: Supplementary file 1 — Supplementary Information. [file 41598_2023_42442_MOESM1_ESM.pdf]

Computational redesign of Beta-27 Fab with substantially better predicted binding affinity to the SARS-CoV-2 Omicron variant than human ACE2 receptor

Wantanee Treewattanawong[1], Thassanai Sitthiyotha[1], Surasak Chunsrivirot[1,]*

[1]Structural and Computational Biology Research Unit, Department of Biochemistry, Faculty of Science, Chulalongkorn University, Pathumwan, Bangkok 10330, Thailand.

[*]Surasak Chunsrivirot
Email: surasak.ch@chula.ac.th

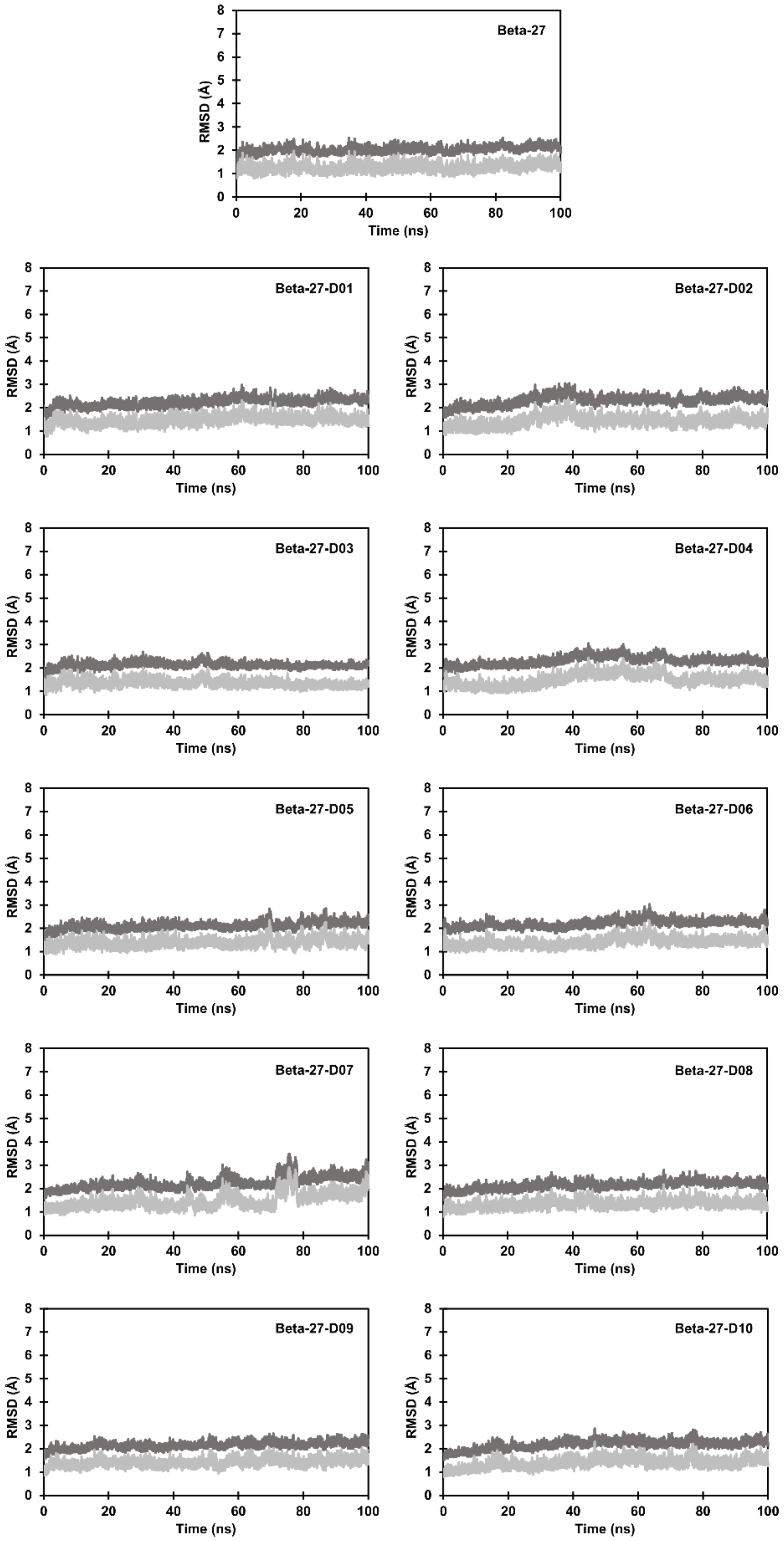

Figure S1. RMSD plots of Beta-27 Fab and designed Beta-27 Fabs in complex with SARS-CoV-2-RBD Omicron variant. The RMSD values of all atoms and backbone atoms (residue 1 to 115 of the heavy chain and residue 1 to 106 of the light chain of Beta-27 Fab and designed Beta-27 Fabs, and all residues of the SARS-CoV-2-RBD Omicron variant) are shown in dark gray and gray, respectively.

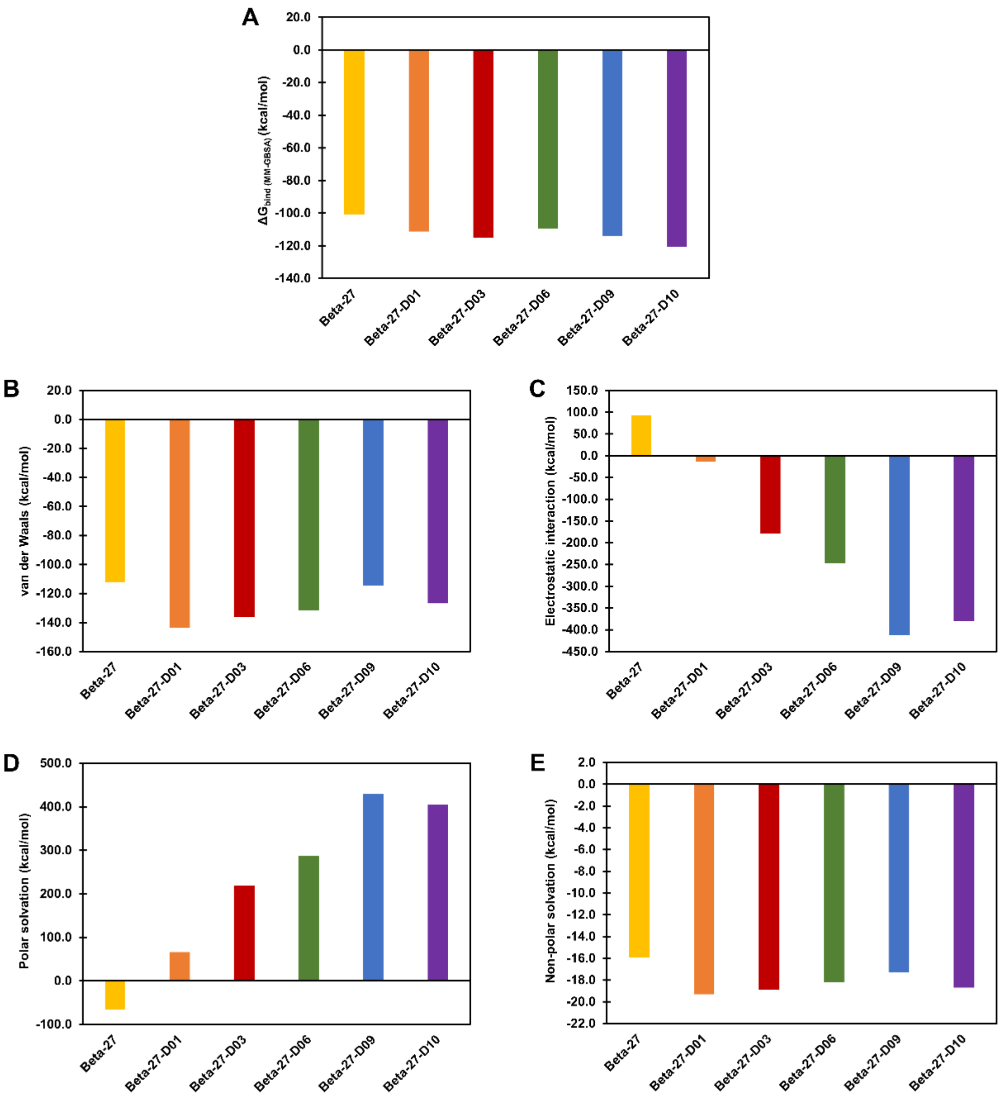

Figure S2. Binding free energy components of Beta-27, Beta-27-D01, Beta-27-D03, Beta-27-D06, Beta-27-D09, and Beta-27-D10 Fabs binding to SARS-CoV-2-RBD Omicron variant. (A) $\Delta G_{bind\ (MM\text{-}GBSA)}$, (B) van der Waals energy, (C) electrostatic interaction, (D) polar solvation, and (E) non-polar solvation.

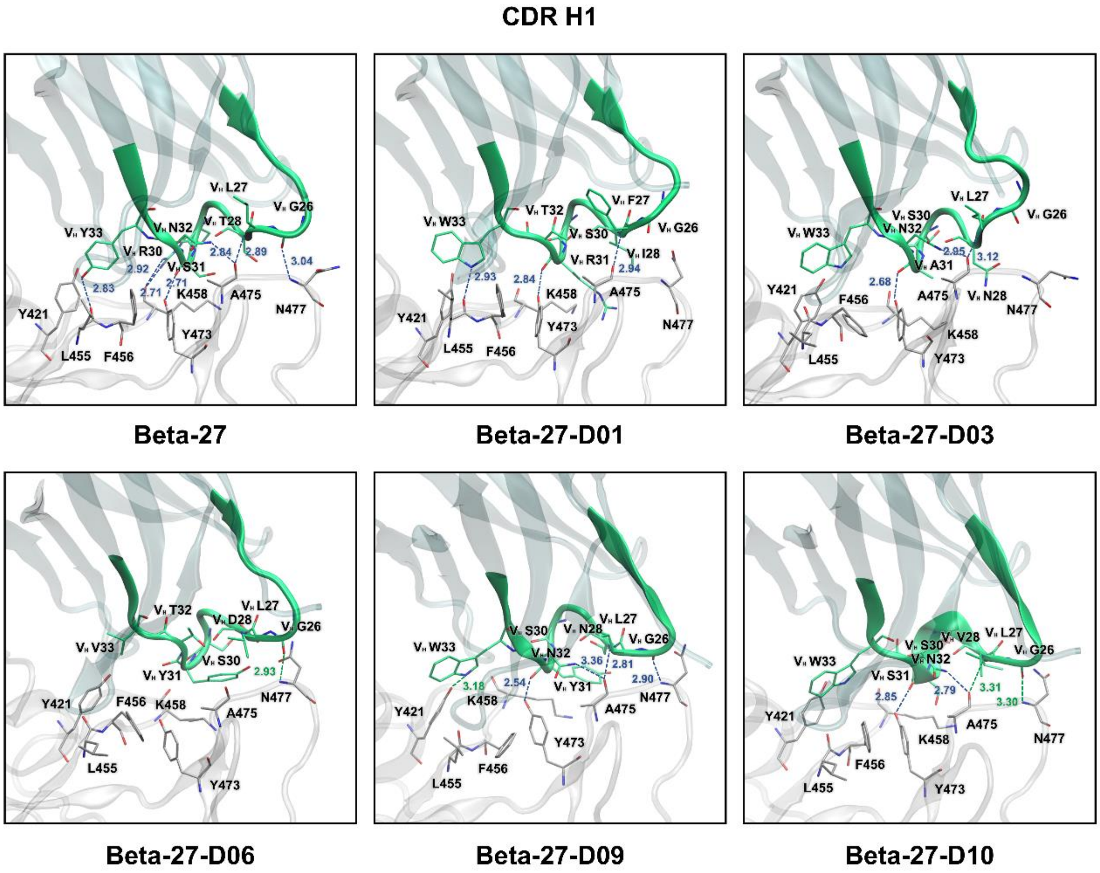

Figure S3. Key binding interactions between SARS-CoV-2-RBD Omicron variant and CDR H1 of the heavy chain of Beta-27, Beta-27-D01, Beta-27-D03, Beta-27-D06, Beta-27-D09, and Beta-27-D10 Fabs. Strong and medium H-bonds are shown in blue and green dashed lines, respectively. Distance (Å) of strong and medium hydrogen bonds are labeled in blue and green, respectively.

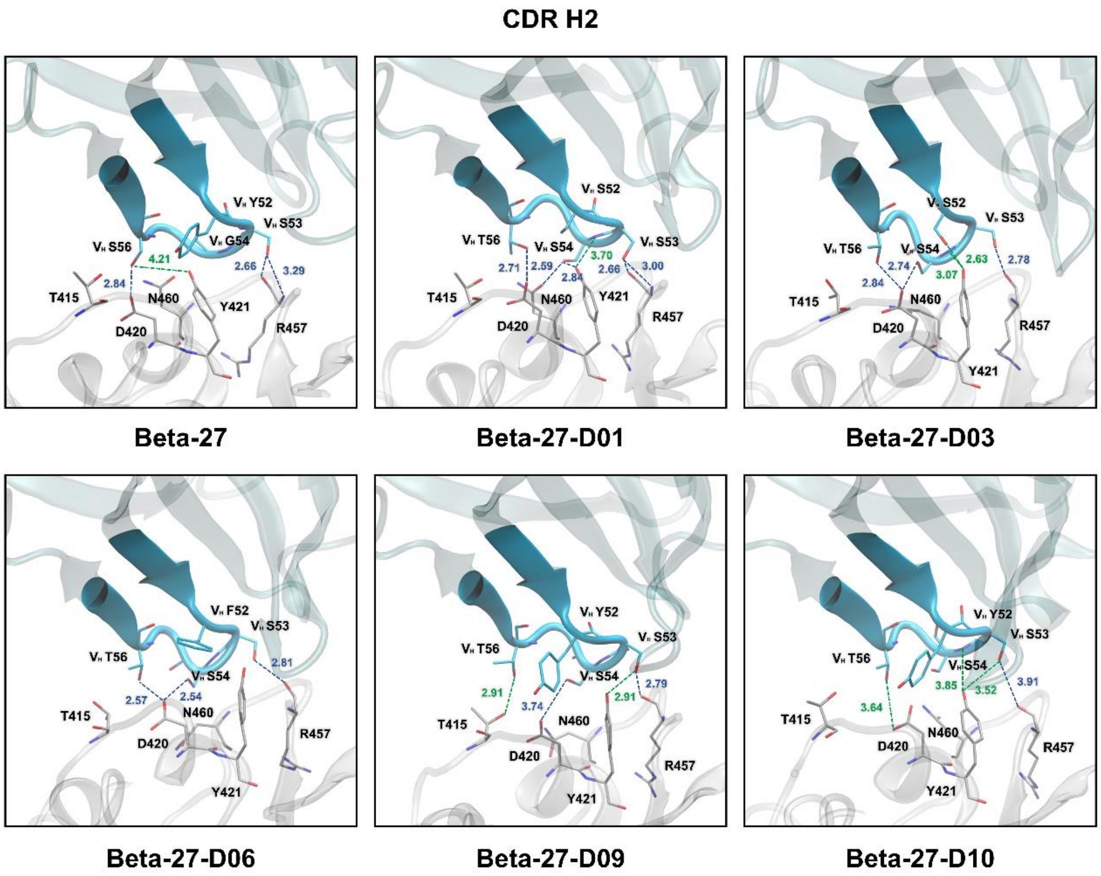

Figure S4. Key binding interactions between SARS-CoV-2-RBD Omicron variant and CDR H2 of the heavy chain of Beta-27, Beta-27-D01, Beta-27-D03, Beta-27-D06, Beta-27-D09, and Beta-27-D10 Fabs. Strong and medium H-bonds are shown in blue and green dashed lines, respectively. Distance (Å) of strong and medium hydrogen bonds are labeled in blue and green, respectively.

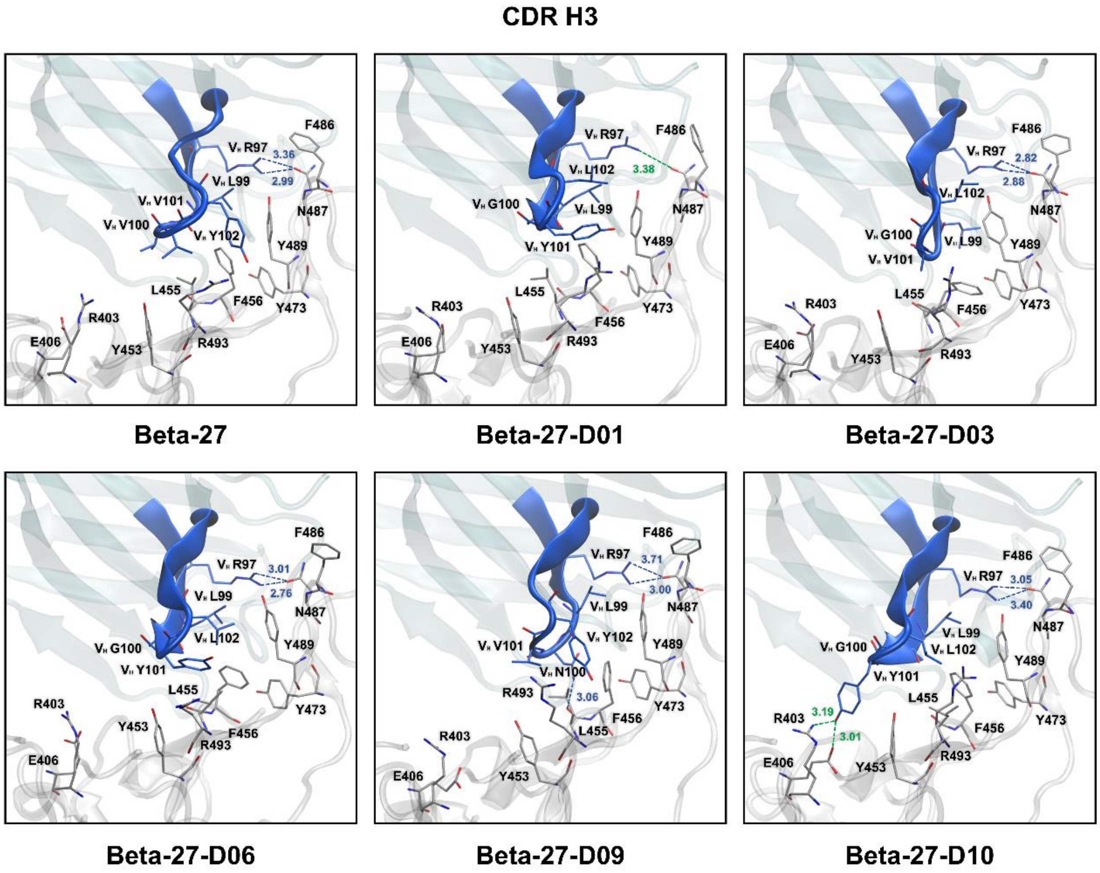

Figure S5. Key binding interactions between SARS-CoV-2-RBD Omicron variant and CDR H3 of the heavy chain of Beta-27, Beta-27-D01, Beta-27-D03, Beta-27-D06, Beta-27-D09, and Beta-27-D10 Fabs. Strong and medium H-bonds are shown in blue and green dashed lines, respectively. Distance (Å) of strong and medium hydrogen bonds are labeled in blue and green, respectively.

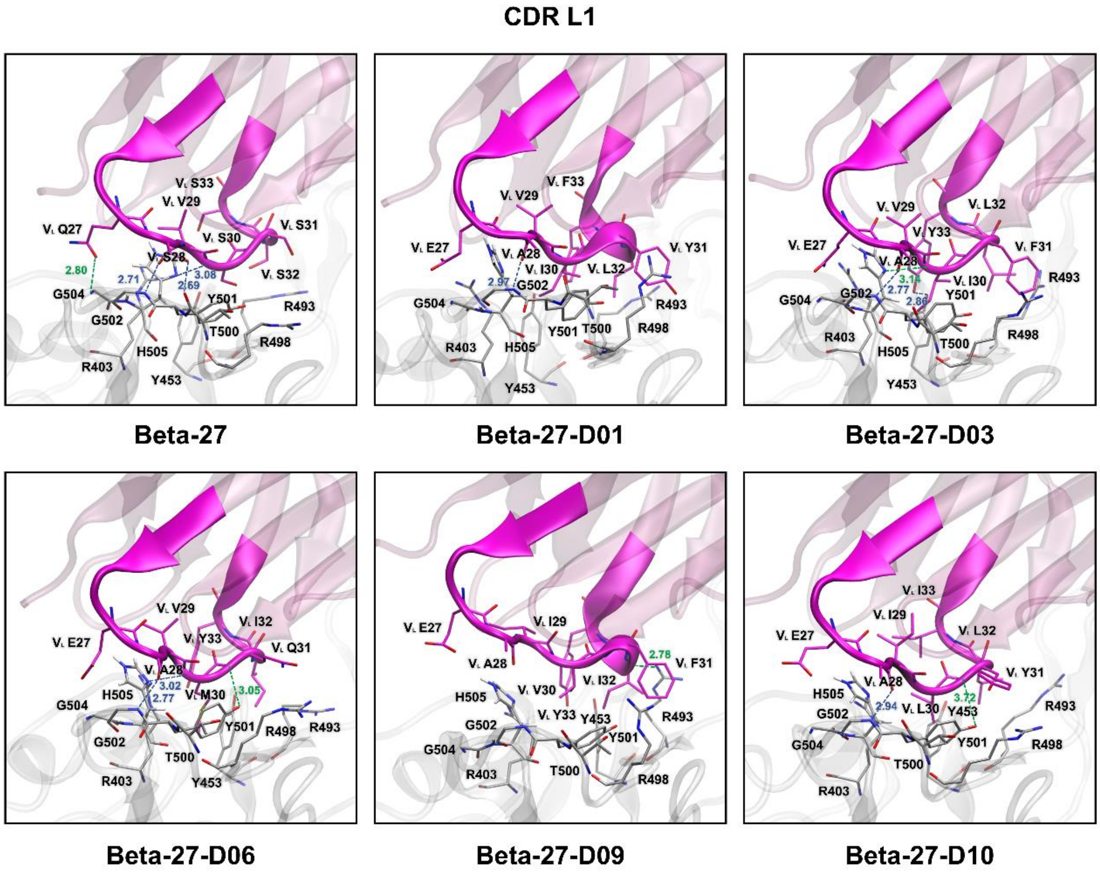

Figure S6. Key binding interactions between SARS-CoV-2-RBD Omicron variant and CDR L1 of the light chain of Beta-27, Beta-27-D01, Beta-27-D03, Beta-27-D06, Beta-27-D09, and Beta-27-D10 Fabs. Strong and medium H-bonds are shown in blue and green dashed lines, respectively. Distance (Å) of strong and medium hydrogen bonds are labeled in blue and green, respectively.

Beta (B.1.351)	TNLCPFGEVFNATRFASVYAWNRKRISNCVADYSVLYNSASFSTFKCYGVSPTKLNDLCF	392
Omicron (B.1.1.529)	TNLCPFDEVFNATRFASVYAWNRKRISNCVADYSVLYNLAPFFTFKCYGVSPTKLNDLCF	392
Omicron (BA.1)	TNLCPFDEVFNATRFASVYAWNRKRISNCVADYSVLYNLAPFFTFKCYGVSPTKLNDLCF	392
Omicron (BA.1.1)	TNLCPFDEVFNATKFASVYAWNRKRISNCVADYSVLYNLAPFFTFKCYGVSPTKLNDLCF	392
Omicron (BA.2)	TNLCPFDEVFNATRFASVYAWNRKRISNCVADYSVLYNFAPFFAFKCYGVSPTKLNDLCF	392
Omicron (BA.2.12.1)	TNLCPFDEVFNATRFASVYAWNRKRISNCVADYSVLYNFAPFFAFKCYGVSPTKLNDLCF	392
Omicron (BA.2.75)	TNLCPFHEVFNATRFASVYAWNRKRISNCVADYSVLYNFAPFFAFKCYGVSPTKLNDLCF	392
Omicron (BA.3)	TNLCPFDEVFNATRFASVYAWNRKRISNCVADYSVLYNFAPFFTFKCYGVSPTKLNDLCF	392
Omicron (BA.4/5)	TNLCPFDEVFNATRFASVYAWNRKRISNCVADYSVLYNFAPFFAFKCYGVSPTKLNDLCF	392
Omicron (BQ.1.1)	TNLCPFDEVFNATTFASVYAWNRKRISNCVADYSVLYNFAPFFAFKCYGVSPTKLNDLCF	392
Omicron (XBB.1)	TNLCPFHEVFNATTFASVYAWNRKRISNCVADYSVIYNFAPFFAFKCYGVSPTKLNDLCF	392

Beta (B.1.351)	TNVYADSFVIRGDEVRQIAPGQTGNIADYNYKLPDDFTGCVIAWNSNNLDSKVGGNYNYL	452
Omicron (B.1.1.529)	TNVYADSFVIRGDEVRQIAPGQTGNIADYNYKLPDDFTGCVIAWNSNKLDSKVSGNYNYL	452
Omicron (BA.1)	TNVYADSFVIRGDEVRQIAPGQTGNIADYNYKLPDDFTGCVIAWNSNKLDSKVSGNYNYL	452
Omicron (BA.1.1)	TNVYADSFVIRGDEVRQIAPGQTGNIADYNYKLPDDFTGCVIAWNSNKLDSKVSGNYNYL	452
Omicron (BA.2)	TNVYADSFVIRGNEVSQIAPGQTGNIADYNYKLPDDFTGCVIAWNSNKLDSKVGGNYNYL	452
Omicron (BA.2.12.1)	TNVYADSFVIRGNEVSQIAPGQTGNIADYNYKLPDDFTGCVIAWNSNKLDSKVGGNYNYQ	452
Omicron (BA.2.75)	TNVYADSFVIRGNEVSQIAPGQTGNIADYNYKLPDDFTGCVIAWNSNKLDSKVSGNYNYL	452
Omicron (BA.3)	TNVYADSFVIRGNEVRQIAPGQTGNIADYNYKLPDDFTGCVIAWNSNKLDSKVSGNYNYL	452
Omicron (BA.4/5)	TNVYADSFVIRGNEVSQIAPGQTGNIADYNYKLPDDFTGCVIAWNSNKLDSKVGGNYNYR	452
Omicron (BQ.1.1)	TNVYADSFVIRGNEVSQIAPGQTGNIADYNYKLPDDFTGCVIAWNSNKLDSTVGGNYNYR	452
Omicron (XBB.1)	TNVYADSFVIRGNEVSQIAPGQTGNIADYNYKLPDDFTGCVIAWNSNKLDSKPSGNYNYL	452

Beta (B.1.351)	YRLFRKSNLKPFERDISTEIYQAGSTPCNGVKGFNCYFPLQSYGFQPTYGVGYQPYRVVV	512
Omicron (B.1.1.529)	YRLFRKSNLKPFERDISTEIYQAGNKPCNGVAGFNCYFPLRSYSFRPTYGVGHQPYRVVV	512
Omicron (BA.1)	YRLFRKSNLKPFERDISTEIYQAGNKPCNGVAGFNCYFPLRSYSFRPTYGVGHQPYRVVV	512
Omicron (BA.1.1)	YRLFRKSNLKPFERDISTEIYQAGNKPCNGVAGFNCYFPLRSYSFRPTYGVGHQPYRVVV	512
Omicron (BA.2)	YRLFRKSNLKPFERDISTEIYQAGNKPCNGVAGFNCYFPLRSYGFRPTYGVGHQPYRVVV	512
Omicron (BA.2.12.1)	YRLFRKSNLKPFERDISTEIYQAGNKPCNGVAGFNCYFPLRSYGFRPTYGVGHQPYRVVV	512
Omicron (BA.2.75)	YRLFRKSNLKPFERDISTEIYQAGNKPCNGVAGFNCYFPLQSYGFRPTYGVGHQPYRVVV	512
Omicron (BA.3)	YRLFRKSNLKPFERDISTEIYQAGNKPCNGVAGFNCYFPLRSYGFRPTYGVGHQPYRVVV	512
Omicron (BA.4/5)	YRLFRKSNLKPFERDISTEIYQAGNKPCNGVAGVNCYFPLQSYGFRPTYGVGHQPYRVVV	512
Omicron (BQ.1.1)	YRLFRKSKLKPFERDISTEIYQAGNKPCNGVAGVNCYFPLQSYGFRPTYGVGHQPYRVVV	512
Omicron (XBB.1)	YRLFRKSKLKPFERDISTEIYQAGNKPCNGVAGSNCYSPLQSYGFRPTYGVGHQPYRVVV	512

Beta (B.1.351)	LSFELLHAPATVCG	526
Omicron (B.1.1.529)	LSFELLHAPATVCG	526
Omicron (BA.1)	LSFELLHAPATVCG	526
Omicron (BA.1.1)	LSFELLHAPATVCG	526
Omicron (BA.2)	LSFELLHAPATVCG	526
Omicron (BA.2.12.1)	LSFELLHAPATVCG	526
Omicron (BA.2.75)	LSFELLHAPATVCG	526
Omicron (BA.3)	LSFELLHAPATVCG	526
Omicron (BA.4/5)	LSFELLHAPATVCG	526
Omicron (BQ.1.1)	LSFELLHAPATVCG	526
Omicron (XBB.1)	LSFELLHAPATVCG	526

Figure S7. Sequence alignment of RBDs of Beta, Omicron and Omicron subvariants that have crystal structures available in the protein databank. Residues involved in binding to Beta 27 Fab (7PS1) were determined by PISA and are highlighted in black. Additionally mutated residues of RBDs of Omicron and Omicron subvariants are highlighted in gray.

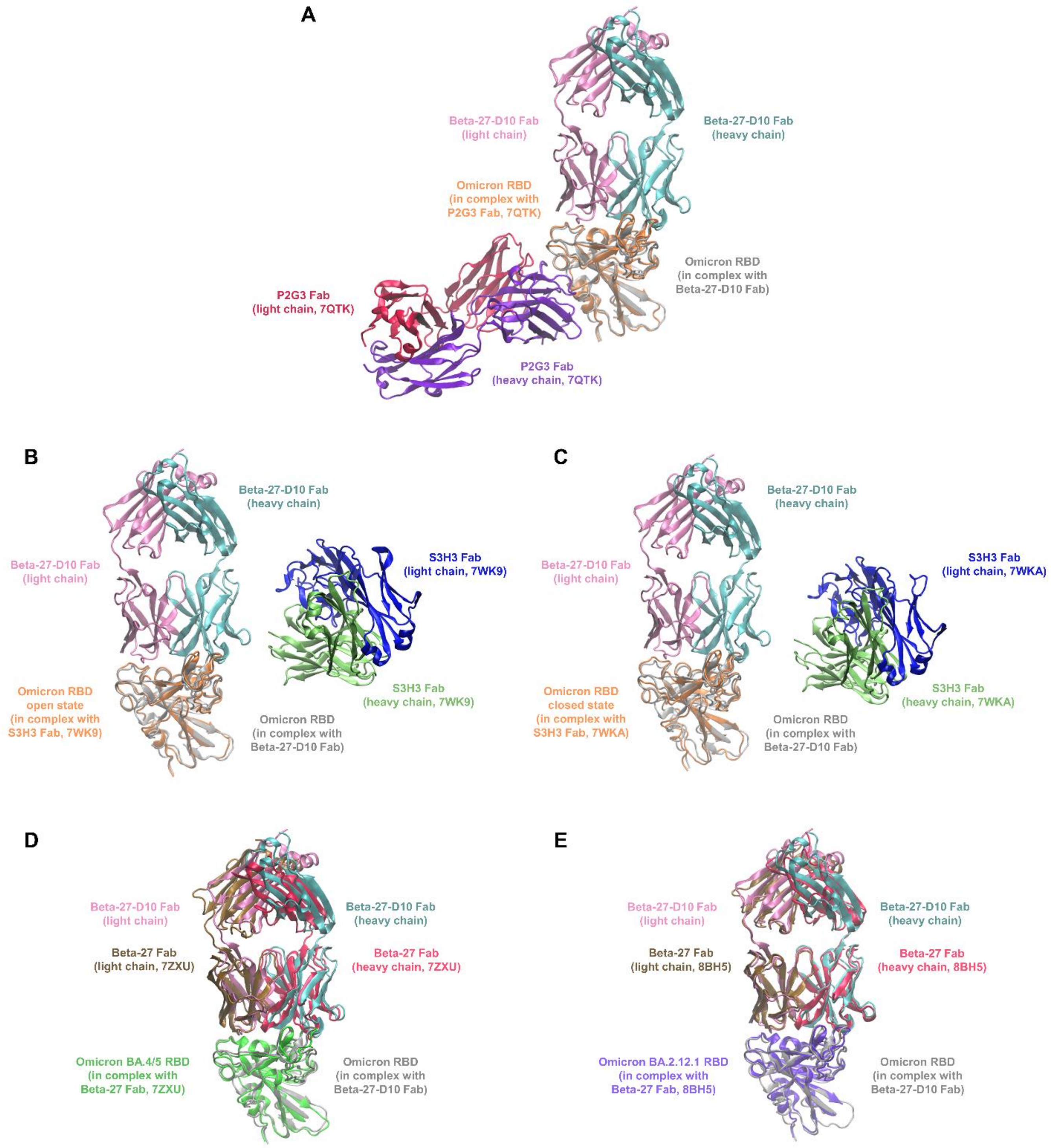

Figure S8. Superimposition of the structures of Beta-27-D10 Fab/Omicron RBD complex and (A) P2G3 Fab/Omicron RBD complex (7QTK), (B) S2H3 Fab/open-state Omicron RBD complex (7WK9), (C) S2H3 Fab/close-state Omicron RBD complex (7WKA), (D) Beta-27 Fab/Omicron BA.4/5 RBD complex (7ZXU) and (E) Beta-27 Fab/Omicron BA.2.12.1 RBD complex (8BH5).

A

Heavy chain

```
Beta-27 Fab       EVQLLESGGGLVQPGGSLRLSCAASGLTVRSNYMNWVRQAPGKGLEWVSLI-YSGGSTFY   59
Beta-27-D01 Fab   EVQLLESGGGLVQPGGSLRLSCTASGFILSRTWLTWVRQAPGKGLEWVSLI-SSSGTTFY   59
Beta-27-D03 Fab   EVQLLESGGGLVQPGGSLRLSCVASGLNLSANWWTWVRQAPGKGLEWVSLI-SSSGTTFY   59
Beta-27-D06 Fab   EVQLLESGGGLVQPGGSLRLSCIVSGLDISYTVMTWVRQAPGKGLEWVSVI-FSSGTTYY   59
Beta-27-D09 Fab   EVQLLESGGGLVQPGGSLRLSCTASGLNISYNWMNWVRQAPGKGLEWVSLI-YSSGTTYY   59
Beta-27-D10 Fab   EVQLLESGGGLVQPGGSLRLSCTASGLVVSSNWLSWVRQAPGKGLEWVSLI-YSSGTTFY   59
P2G3 Fab          EVQLVESGGGLVQPGRSLRLSCAASGFRFDDYALHWVRQAPEKGLEWVSGISWNSNNIGY   60
S3H3 Fab          QVQLQQPGAELVRPGASVKLSCKASGYSFTRFWMNWVKQRPGQGLEWIGMIHPSDSETRL   60

Beta-27 Fab       ADSVKGRFTISRHDSKNTLYLQMNSLRAEDTAVYYCARDLVV-------YGMDVWGQGTT   112
Beta-27-D01 Fab   ADSVKGRFTISRHDSKNTLYLQMNSLRAEDTAVYYCARLLGY-------LGMDVWGQGTT   112
Beta-27-D03 Fab   ADSVKGRFTISRHDSKNTLYLQMNSLRAEDTAVYYCARLLGV-------LGMDVWGQGTT   112
Beta-27-D06 Fab   ADSVKGRFTISRHDSKNTLYLQMNSLRAEDTAVYYCARLLGY-------LGMDVWGQGTT   112
Beta-27-D09 Fab   ADSVKGRFTISRHDSKNTLYLQMNSLRAEDTAVYYCARLLNV-------YGMDVWGQGTT   112
Beta-27-D10 Fab   ADSVKGRFTISRHDSKNTLYLQMNSLRAEDTAVYYCARLLGY-------LGMDVWGQGTT   112
P2G3 Fab          AESVKGRFTISRDTAKKSLYLQMNDLRAEDTALYYCVKDRHYDSSGYFVNGFDIWGQGTL   120
S3H3 Fab          NQKFKDKATLTVDKSSTTAYMQLSSPTSEDSAVYYCARKD-YDYDA----WFAYWGQGTL   115

Beta-27 Fab       VTVSSASTKGPSVFPLAPSSKSTSGGTAALGCLVKDYFPEPVTVSWNSGALTSGVHTFPA   172
Beta-27-D01 Fab   VTVSSASTKGPSVFPLAPSSKSTSGGTAALGCLVKDYFPEPVTVSWNSGALTSGVHTFPA   172
Beta-27-D03 Fab   VTVSSASTKGPSVFPLAPSSKSTSGGTAALGCLVKDYFPEPVTVSWNSGALTSGVHTFPA   172
Beta-27-D06 Fab   VTVSSASTKGPSVFPLAPSSKSTSGGTAALGCLVKDYFPEPVTVSWNSGALTSGVHTFPA   172
Beta-27-D09 Fab   VTVSSASTKGPSVFPLAPSSKSTSGGTAALGCLVKDYFPEPVTVSWNSGALTSGVHTFPA   172
Beta-27-D10 Fab   VTVSSASTKGPSVFPLAPSSKSTSGGTAALGCLVKDYFPEPVTVSWNSGALTSGVHTFPA   172
P2G3 Fab          VTVSAASTKGPSVFPLAPSSKSTSGGTAALGCLVKDYFPEPVTVSWNSGALTSGVHTFPA   180
S3H3 Fab          VTVSAAKTTPPSVYPLAPGSAAQTNSMVTLGCLVKGYFPEPVTVTWNSGSLSSGVHTFPA   175

Beta-27 Fab       VLQSSGLYSLSSVVTVPSSSLGTQTYICNVNHKPSNTKVDKKVEPKSCDK   222
Beta-27-D01 Fab   VLQSSGLYSLSSVVTVPSSSLGTQTYICNVNHKPSNTKVDKKVEPKSCDK   222
Beta-27-D03 Fab   VLQSSGLYSLSSVVTVPSSSLGTQTYICNVNHKPSNTKVDKKVEPKSCDK   222
Beta-27-D06 Fab   VLQSSGLYSLSSVVTVPSSSLGTQTYICNVNHKPSNTKVDKKVEPKSCDK   222
Beta-27-D09 Fab   VLQSSGLYSLSSVVTVPSSSLGTQTYICNVNHKPSNTKVDKKVEPKSCDK   222
Beta-27-D10 Fab   VLQSSGLYSLSSVVTVPSSSLGTQTYICNVNHKPSNTKVDKKVEPKSCDK   222
P2G3 Fab          VLQSSGLYSLSSVVTVPSSSLGTQTYICNVNHKPSNTKVDKRVEPKSC--   228
S3H3 Fab          VLQS-DLYTLSSSVTVPSSTWPSETVTCNVAHPASSTKVDKKI-------   217
```

B

Light chain

```
Beta-27 Fab       DIQMTQSPGTLSLSPGERATLSCRASQSVSS---SSLAWYQQKHGQAPRLLIYGTSSRAT   57
Beta-27-D01 Fab   DIQMTQSPGTLSLSPGERATLSCKFSEAVIY---LFFCWYQQKHGQAPRLLIYSTSYLYP   57
Beta-27-D03 Fab   DIQMTQSPGTLSLSPGERATLSCQFSEAVIF---LYVAWYQQKHGQAPRLLIYETSKLYP   57
Beta-27-D06 Fab   DIQMTQSPGTLSLSPGERATLSCKSSEAVMQ---IYVAWYQQKHGQAPRLLIYATTYLAP   57
Beta-27-D09 Fab   DIQMTQSPGTLSLSPGERATLSCKSSEAIVF---IYWCWYQQKHGQAPRLLIYDTSLLHP   57
Beta-27-D10 Fab   DIQMTQSPGTLSLSPGERATLSCKFSEAILY---LIVCWYQQKHGQAPRLLIYETSKLHE   57
P2G3 Fab          DIQLTQSPSFLSASVGDRVTVTCRASQGIS----SYVAWYQQKAGKAPTLLIYTASTLQS   56
S3H3 Fab          DIVLTQSPASLAVSLGQRATISCRASKSVSASVYSYMHWYQQKPGQPPKLLIYLASSLES   60

Beta-27 Fab       GIPDRFSGSGSGTDFTLTISGLEPEDFAVYYCQQYGSSP-LFGGGTKVEIKRTVAAPSVF   116
Beta-27-D01 Fab   GIPDRFSGSGSGTDFTLTISGLEPEDFAVYYCMYYTQVP-YFGGGTKVEIKRTVAAPSVF   116
Beta-27-D03 Fab   GIPDRFSGSGSGTDFTLTISGLEPEDFAVYYCVFYTQVP-YFGGGTKVEIKRTVAAPSVF   116
Beta-27-D06 Fab   GIPDRFSGSGSGTDFTLTISGLEPEDFAVYYCCLYGEVP-YFGGGTKVEIKRTVAAPSVF   116
Beta-27-D09 Fab   GIPDRFSGSGSGTDFTLTISGLEPEDFAVYYCLMIGEVP-QFGGGTKVEIKRTVAAPSVF   116
Beta-27-D10 Fab   GIPDRFSGSGSGTDFTLTISGLEPEDFAVYYCVMFTEVP-YFGGGTKVEIKRTVAAPSVF   116
P2G3 Fab          GVPSRFSGSGSGTEFTLTISSLQPEDFATYYCQQLHSYPVTFGQGTRLDIERTVAAPSVF   116
S3H3 Fab          GVPARFSGSGSGTDFTLNIHPVEEEDAATYYCHHSRELPPAFGGGTKLEIKRADAAPTVS   120

Beta-27 Fab       IFPPSDEQLKSGTASVVCLLNNFYPREAKVQWKVDNALQSGNSQESVTEQDSKDSTYSLS   176
Beta-27-D01 Fab   IFPPSDEQLKSGTASVVCLLNNFYPREAKVQWKVDNALQSGNSQESVTEQDSKDSTYSLS   176
Beta-27-D03 Fab   IFPPSDEQLKSGTASVVCLLNNFYPREAKVQWKVDNALQSGNSQESVTEQDSKDSTYSLS   176
Beta-27-D06 Fab   IFPPSDEQLKSGTASVVCLLNNFYPREAKVQWKVDNALQSGNSQESVTEQDSKDSTYSLS   176
Beta-27-D09 Fab   IFPPSDEQLKSGTASVVCLLNNFYPREAKVQWKVDNALQSGNSQESVTEQDSKDSTYSLS   176
Beta-27-D10 Fab   IFPPSDEQLKSGTASVVCLLNNFYPREAKVQWKVDNALQSGNSQESVTEQDSKDSTYSLS   176
P2G3 Fab          IFPPSDEQLKSGTASVVCLLNNFYPREAKVQWKVDNALQSGNSQESVTEQDSKDSTYSLS   176
S3H3 Fab          IFPPSSEQLTSGGASVVCFLNNFYPKDINVKWKIDGSERQNGVLNSWTDQDSKDSTYSMS   180

Beta-27 Fab       STLTLSKADYEKHKVYACEVTHQGLSSPVTKSFNRGEC   214
Beta-27-D01 Fab   STLTLSKADYEKHKVYACEVTHQGLSSPVTKSFNRGEC   214
Beta-27-D03 Fab   STLTLSKADYEKHKVYACEVTHQGLSSPVTKSFNRGEC   214
Beta-27-D06 Fab   STLTLSKADYEKHKVYACEVTHQGLSSPVTKSFNRGEC   214
Beta-27-D09 Fab   STLTLSKADYEKHKVYACEVTHQGLSSPVTKSFNRGEC   214
Beta-27-D10 Fab   STLTLSKADYEKHKVYACEVTHQGLSSPVTKSFNRGEC   214
P2G3 Fab          STLTLSKADYEKHKVYACEVTHQGLSSPVTKSFNRGEC   214
S3H3 Fab          STLTLTKDEYERHNSYTCEATHKTSTSPIVKSFNR---   215
```

Figure S9. Sequence alignment of the heavy chain (A) and light chain (B) of Beta-27 Fab, Beta-27-D01 Fab, Beta-27-D03 Fab, Beta-27-D06 Fab, Beta-27-D09 Fab, Beta-27-D10 Fab, P2G3 Fab and S3H3 Fab.

Table S1. Hydrogen bond occupations of Beta-27 Fab involved in SARS-CoV-2-RBD Omicron variant binding.

CDRs	Acceptor	DonorH	Donor	Hydrogen bond occupancy (%)
-	D1(L)@OD1	R408@HH12	R408@NH1	29.5
	D1(L)@OD2	R408@HH12	R408@NH1	32.8
	D1(L)@OD1	R408@HH22	R408@NH2	34.7
	D1(L)@OD2	R408@HH22	R408@NH2	39.5
H1	G26@O	N477@H	N477@N	92.1
	G26@O	N477@HD22	N477@ND2	6.3
	A475@O	T28@H	T28@N	92.0
	K458@O	R30@HH12	R30@NH1	96.9
	K458@O	R30@HH22	R30@NH2	96.8
	S31@O	Y473@HH	Y473@OH	99.4
	Q474@O	S31@HG	S31@OG	7.5
	A475@O	N32@HD21	N32@ND2	99.2
	Y33@OH	N417@HD22	N417@ND2	9.5
	L455@O	Y33@HH	Y33@OH	96.2
H2	Y52@OH	N417@H	N417@N	12.0
	S53@OG	R457@H	R457@N	78.1
	R457@O	S53@HG	S53@OG	98.8
	Y421@OH	G54@H	G54@N	27.4
	N460@OD1	G55@H	G55@N	47.7
	G55@O	N460@HD21	N460@ND2	11.1
	Y421@OH	S56@H	S56@N	32.7
	T415@OG1	S56@HG	S56@OG	14.8
	D420@OD1	S56@HG	S56@OG	98.6
	S56@OG	Y421@HH	Y421@OH	53.7
	S56@OG	N460@HD21	N460@ND2	29.0
H3	N487@OD1	R97@HH12	R97@NH1	99.9
	N487@OD1	R97@HH22	R97@NH2	75.7
	Y102@OH	R493@HH21	R493@NH2	16.2
L1	Q27@OE1	G504@H	G504@N	54.7
	S28@O	G502@H	G502@N	94.0
	T500@O	S28@HG	S28@OG	88.6
	H505@ND1	S30@H	S30@N	80.1
	Y501@OH	S30@HG	S30@OG	14.0
	S32@OG	R493@HH11	R493@NH1	6.6

Table S2. Hydrogen bond occupations of Beta-27-D01 Fab involved in SARS-CoV-2-RBD Omicron variant binding.

CDRs	Acceptor	DonorH	Donor	Hydrogen bond occupancy (%)
-	D1(L)@OD2	R408@HH22	R408@NH2	6.2
	G67(L)@O	R498@HH22	R498@NH2	6.8
H1	S25@O	N477@HD21	N477@ND2	23.5
	G26@O	N477@H	N477@N	29.3
	G26@O	N477@HD22	N477@ND2	8.2
	G26@O	N487@HD21	N487@ND2	29.2
	A475@O	I28@H	I28@N	97.5
	Q474@O	R31@HH11	R31@NH1	49.1
	R31@O	Y473@HH	Y473@OH	99.9
	L455@O	W33@HE1	W33@NE1	90.7
H2	Y421@OH	S53@HG	S53@OG	26.4
	S53@OG	R457@H	R457@N	95.3
	R457@O	S53@HG	S53@OG	91.2
	Y421@OH	S54@H	S54@N	70.9
	S54@OG	Y421@HH	Y421@OH	99.8
	N460@OD1	S54@HG	S54@OG	99.8
	D420@OD2	T56@HG1	T56@OG1	95.7
H3	N487@OD1	R97@HH12	R97@NH1	56.0
	N487@OD1	R97@HH22	R97@NH2	10.8
L1	E27@OE1	G504@H	G504@N	18.5
	E27@OE1	H505@HE2	H505@NE2	20.7
	E27@OE2	G504@H	G504@N	26.9
	E27@OE2	H505@HE2	H505@NE2	9.9
	A28@O	G502@H	G502@N	95.0
	H505@ND1	I30@H	I30@N	17.9
	Y501@OH	Y31@H	Y31@N	9.4
	Y31@OH	S496@HG	S496@OG	35.4
	S496@OG	Y31@HH	Y31@OH	16.1
L3	Q94@OE1	N417@H	N417@N	99.1
	Q94@OE1	N417@HD22	N417@ND2	96.6

Table S3. Hydrogen bond occupations of Beta-27-D03 Fab involved in SARS-CoV-2-RBD Omicron variant binding.

CDRs	Acceptor	DonorH	Donor	Hydrogen bond occupancy (%)
-	D1(L)@OD1	R408@HH12	R408@NH1	17.3
	D1(L)@OD1	R408@HH22	R408@NH2	15.9
	D1(L)@OD2	R408@HH12	R408@NH1	14.6
	D1(L)@OD2	R408@HH22	R408@NH2	21.2
H1	G26@O	N477@HD21	N477@ND2	8.8
	A475@O	N28@H	N28@N	85.4
	G476@O	N28@HD21	N28@ND2	43.5
	Q474@O	N28@HD22	N28@ND2	16.5
	A31@O	Y473@HH	Y473@OH	99.5
	A475@O	N32@HD21	N32@ND2	99.5
H2	S52@OG	Y421@HH	Y421@OH	50.6
	S53@OG	R457@H	R457@N	8.2
	R457@O	S53@HG	S53@OG	99.2
	Y421@OH	S54@H	S54@N	65.7
	D420@OD2	S54@HG	S54@OG	82.1
	S54@OG	Y421@HH	Y421@OH	12.4
	T415@OG1	T56@HG1	T56@OG1	5.6
	D420@OD1	T56@HG1	T56@OG1	14.2
	D420@OD2	T56@HG1	T56@OG1	78.2
H3	N487@OD1	R97@HH12	R97@NH1	99.9
	N487@OD1	R97@HH22	R97@NH2	98.3
L1	E27@OE1	V503@H	V503@N	7.0
	E27@OE1	G504@H	G504@N	27.0
	E27@OE2	G504@H	G504@N	31.0
	A28@O	G502@H	G502@N	99.8
	H505@ND1	I30@H	I30@N	70.6
	Y453@OH	Y33@HH	Y33@OH	89.8
L2	E51@OE1	R493@HH12	R493@NH1	77.8
	E51@OE1	R493@HH22	R493@NH2	32.6
	E51@OE2	R493@HH12	R493@NH1	47.7
	E51@OE2	R493@HH22	R493@NH2	78.2
L3	Q409@NE2	Q94@HE21	Q94@NE2	29.2
	N417@OD1	Q94@HE21	Q94@NE2	17.3
	E406@OE1	Q94@HE22	Q94@NE2	27.5
	E406@OE2	Q94@HE22	Q94@NE2	17.7
	Q94@OE1	N417@H	N417@N	83.9

Table S4. Hydrogen bond occupations of Beta-27-D06 Fab involved in SARS-CoV-2-RBD Omicron variant binding.

CDRs	Acceptor	DonorH	Donor	Hydrogen bond occupancy (%)
H1	G26@O	N477@H	N477@N	66.0
	L27@O	N477@HD21	N477@ND2	5.4
	A475@O	D28@H	D28@N	28.1
	Y31@O	Y473@HH	Y473@OH	37.4
	Q474@O	Y31@HH	Y31@OH	15.6
H2	Y421@OH	S53@H	S53@N	19.4
	S53@OG	Y421@HH	Y421@OH	46.6
	S53@OG	R457@H	R457@N	5.1
	R457@O	S53@HG	S53@OG	99.2
	Y421@OH	S54@H	S54@N	7.4
	D420@OD2	S54@HG	S54@OG	99.3
	D420@OD2	T56@HG1	T56@OG1	99.9
	T415@O	Y58@HH	Y58@OH	5.3
	Y58@OH	T415@HG1	T415@OG1	11.7
H3	A475@O	R97@HH12	R97@NH1	6.1
	N487@OD1	R97@HH12	R97@NH1	96.8
	N487@OD1	R97@HH22	R97@NH2	90.5
	Y489@OH	R97@HH22	R97@NH2	13.4
	Y101@OH	R493@HH11	R493@NH1	28.7
L1	A28@O	G502@H	G502@N	99.5
	H505@ND1	M30@H	M30@N	87.2
	Y501@OH	Q31@H	Q31@N	63.4
	Q31@OE1	R498@HE	R498@NE	8.9
	Q31@OE1	R498@HH21	R498@NH2	7.7
	Y33@OH	R403@HE	R403@NE	14.6
	E406@OE2	Y33@HH	Y33@OH	5.3
	Y453@OH	Y33@HH	Y33@OH	27.1
L2	Y54@OH	R493@HH12	R493@NH1	15.4
L3	Y92@OH	N417@HD22	N417@ND2	21.0
	E94@OE1	R408@HE	R408@NE	22.1
	E94@OE1	R408@HH12	R408@NH1	23.6
	E94@OE1	R408@HH21	R408@NH2	33.1
	E94@OE1	R408@HH22	R408@NH2	17.7
	E94@OE2	R408@HE	R408@NE	30.8
	E94@OE2	R408@HH12	R408@NH1	20.1
	E94@OE2	R408@HH21	R408@NH2	41.9
	E94@OE2	R408@HH22	R408@NH2	17.6

Table S5. Hydrogen bond occupations of Beta-27-D09 Fab involved in SARS-CoV-2-RBD Omicron variant binding.

CDRs	Acceptor	DonorH	Donor	Hydrogen bond occupancy (%)
-	E1(H)@OE1	K478@HZ1	K478@NZ	15.2
	E1(H)@OE1	K478@HZ2	K478@NZ	12.0
	E1(H)@OE1	K478@HZ3	K478@NZ	13.6
	E1(H)@OE2	K478@HZ1	K478@NZ	14.4
	E1(H)@OE2	K478@HZ2	K478@NZ	13.0
	E1(H)@OE2	K478@HZ3	K478@NZ	13.2
	N76(H)@ND2	N477@HD21	N477@ND2	5.4
H1	G26@O	N477@H	N477@N	82.7
	G26@O	N477@HD22	N477@ND2	6.1
	L27@O	N477@HD21	N477@ND2	6.7
	A475@O	N28@H	N28@N	83.5
	N477@OD1	N28@HD22	N28@ND2	10.2
	Y31@O	Y473@HH	Y473@OH	91.9
	A475@O	N32@HD21	N32@ND2	97.6
	Y421@OH	W33@HE1	W33@NE1	58.4
H2	Y421@OH	S53@H	S53@N	15.7
	S53@OG	Y421@HH	Y421@OH	64.5
	R457@O	S53@HG	S53@OG	92.5
	Y421@OH	S54@H	S54@N	38.2
	S54@O	N460@HD21	N460@ND2	9.8
	D420@OD1	S54@HG	S54@OG	95.3
	T415@OG1	T56@HG1	T56@OG1	70.1
	D420@OD1	T56@HG1	T56@OG1	37.9
H3	N487@OD1	R97@HH12	R97@NH1	99.7
	N487@OD1	R97@HH22	R97@NH2	87.3
	Y489@OH	R97@HH22	R97@NH2	5.2
	L455@O	N100@HD22	N100@ND2	99.4
L1	I32@O	R493@HH21	R493@NH2	59.0
	Y453@OH	Y33@HH	Y33@OH	5.7
L2	D51@OD1	R493@HH12	R493@NH1	48.1
	D51@OD1	R493@HH22	R493@NH2	99.9
	D51@OD2	R493@HH12	R493@NH1	99.1
	D51@OD2	R493@HH22	R493@NH2	49.9
L3	E94@OE1	R408@HH12	R408@NH1	70.6
	E94@OE1	R408@HH22	R408@NH2	69.4
	E94@OE2	R408@HH12	R408@NH1	65.4
	E94@OE2	R408@HH22	R408@NH2	74.2

Table S6. Hydrogen bond occupations of Beta-27-D10 Fab involved in SARS-CoV-2-RBD Omicron variant binding.

CDRs	Acceptor	DonorH	Donor	Hydrogen bond occupancy (%)
-	E1(H)@OE1	K478@HZ1	K478@NZ	8.4
	E1(H)@OE1	K478@HZ2	K478@NZ	10.8
	E1(H)@OE1	K478@HZ3	K478@NZ	6.2
	E1(H)@OE1	N487@HD22	N487@ND2	7.9
	E1(H)@OE2	K478@HZ1	K478@NZ	7.8
	E1(H)@OE2	K478@HZ2	K478@NZ	10.9
	E1(H)@OE2	K478@HZ3	K478@NZ	6.4
H1	G26@O	N477@H	N477@N	74.8
	G26@O	N477@HD22	N477@ND2	7.6
	A475@O	V28@H	V28@N	56.3
	S31@O	Y473@HH	Y473@OH	92.0
	A475@O	N32@HD21	N32@ND2	99.7
H2	Y52@OH	N417@H	N417@N	10.2
	Y421@OH	S53@H	S53@N	25.5
	S53@OG	Y421@HH	Y421@OH	51.5
	Y421@OH	S53@HG	S53@OG	6.5
	S53@OG	R457@H	R457@N	17.5
	R457@O	S53@HG	S53@OG	79.7
	K458@O	S53@HG	S53@OG	5.6
	Y421@OH	S54@H	S54@N	58.1
	D420@OD1	S54@HG	S54@OG	37.8
	D420@OD2	S54@HG	S54@OG	26.6
	S54@OG	Y421@HH	Y421@OH	27.8
	N460@OD1	S54@HG	S54@OG	22.8
	D420@OD1	T56@HG1	T56@OG1	67.7
	D420@OD2	T56@HG1	T56@OG1	28.6
	T56@OG1	N460@HD21	N460@ND2	6.0
H3	N487@OD1	R97@HH12	R97@NH1	99.8
	N487@OD1	R97@HH22	R97@NH2	96.6
	Y489@OH	R97@HH22	R97@NH2	6.1
	Y101@OH	R403@HH21	R403@NH2	55.5
	E406@OE1	Y101@HH	Y101@OH	10.5
	E406@OE2	Y101@HH	Y101@OH	62.9
	D105@OD2	Y489@HH	Y489@OH	5.8
L1	E27@OE1	H505@HE2	H505@NE2	35.0
	E27@OE2	G504@H	G504@N	9.3
	E27@OE2	H505@HE2	H505@NE2	19.3
	A28@O	G502@H	G502@N	99.9
	H505@ND1	L30@H	L30@N	8.6
	Y501@OH	Y31@H	Y31@N	55.2
L2	E51@OE1	R493@HE	R493@NE	47.2
	E51@OE1	R493@HH21	R493@NH2	56.0
	E51@OE2	R493@HE	R493@NE	45.4
	E51@OE2	R493@HH21	R493@NH2	63.1
L3	E94@OE1	R408@HH12	R408@NH1	46.7
	E94@OE1	R408@HH22	R408@NH2	90.2
	E94@OE2	R408@HH12	R408@NH1	50.3
	E94@OE2	R408@HH22	R408@NH2	90.6

Table S7. Pi interactions of Beta-27 Fab, Beta-27-D01 Fab, Beta-27-D03 Fab, Beta-27-D06 Fab, Beta-27-D09 Fab, and Beta-27-D10 Fab involved in SARS-CoV-2-RBD Omicron variant binding.

System	CDRs	Residue that forms pi interaction with SARS-CoV-2-RBD				
		Pi-pi	Cation-pi	Anion-pi	Sigma-pi	Alkyl-pi
Beta-27	-	-	-	E1(H)@OE1-F486	-	-
	H1	Y33-F456	-	-	-	-
	H2	Y52-Y421	-	-	-	-
	H3	Y102-Y489	R97@NH2-F486	-	-	L99-Y489
	L1	-	-	-	-	V29-H505
	L2	-	-	-	-	-
	L3	-	-	-	-	-
Beta-27-D01	-	-	-	-	-	V2(H)-F486
	H1	W33-F456	R31@NH1-Y473	-	-	F27-A475<br>R31-Y473<br>W33-L455
	H2	-	-	-	-	-
	H3	-	R97@NH1-F486<br>R97@NH2-F486<br>Y101-R493@NH1<br>Y101-R493@NH2	-	-	L99-F456<br>L99-Y489<br>Y101-R493
	L1	F33-Y453	Y31-R498@NH1	-	V29@HA-H505	V29-H505<br>I30-Y501<br>I30-H505<br>F33-R493
	L2	-	-	-	-	-
	L3	Y91-H505	-	-	-	-
Beta-27-D03	-	-	-	-	-	V2(H)-F486
	H1	W33-Y421	-	-	-	A31-Y473
	H2	-	-	-	-	-
	H3	-	R97@NH2-F486	-	-	L99-F456<br>L99-Y473<br>L99-Y489
	L1	Y33-H505	F31-R498@NH1<br>F31-R498@NH2	-	V29@HA-H505	V29-H505<br>I30-Y501<br>I30-H505
	L2	-	-	-	-	-
	L3	-	-	-	-	-
Beta-27-D06	-	-	-	-	-	-
	H1	Y31-Y473	Y31-K458@NZ	-	Y31-K458@HE2	Y31-K458<br>Y31-A475<br>V33-F456
	H2	F52-Y421	-	-	-	-
	H3	-	R97@NH2-F486<br>Y101-R493@NH1	-	Y101-R493@HD3	L99-F456<br>L99-Y489<br>Y101-L455<br>Y101-R493<br>L102-Y489
	L1	Y33-H505	Y33-R403@NH1<br>Y33-R403@NH2	-	V29@HA-H505<br>M30@HA-Y501	V29-H505<br>M30-Y501<br>M30-H505
	L2	-	-	-	-	P57-F486
	L3	-	-	-	-	-
Beta-27-D09	-	-	-	-	-	V2(H)-F486
	H1	W33-Y421	Y31-K458@NZ	-	-	Y31-K458
	H2	Y52-Y421	-	-	-	-
	H3	-	R97@NH2-F486	-	-	L99-Y489<br>Y102-L455
	L1	Y33-H505	F31-R498@NH1<br>Y33-R403@NH1<br>Y33-R403@NH2	-	I29@HA-H505	I29-H505<br>V30-Y501<br>V30-H505
	L2	-	-	-	-	P57-F486
	L3	-	-	-	-	-
Beta-27-D10	-	-	-	-	-	V2(H)-F486
	H1	W33-Y421<br>W33-F456	-	-	-	-
	H2	Y52-Y421	-	-	-	-
	H3	Y101-Y453	R97@NH2-F486<br>Y101-R403@NH2	-	-	L99-F456<br>L99-Y489
	L1	Y31-Y501	Y31-R498@NH1	-	I29@HA-H505<br>L30@HA-Y501	I29-H505<br>L30-Y501<br>L30-H505
	L2	-	-	-	-	-
	L3	-	-	-	-	-
